# Trauma inquiry and response in sexual and reproductive health settings: collaborative learning among clinicians

**DOI:** 10.1186/s12978-025-02135-6

**Published:** 2025-09-29

**Authors:** Megha Shankar, Kelsey B Loeliger, Emily Ager, Maud Arnal, Sara Johnson, Zoe Matticks, Emily Nakamura, Hannah Begna, Eleanor Bimla Schwarz

**Affiliations:** 1 Department of Medicine, Division of General Internal Medicine, University of California San Diego, San Diego, CA USA; 2 Department of Obstetrics, Gynecology, and Reproductive Sciences, Division of Complex Family Planning, University of California San Diego, San Diego, CA USA; 3 Department of Emergency Medicine, University of California San Francisco, San Francisco, CA USA; 4 Department of Sociology, University of California San Diego, San Diego, CA USA; 5 La Clinica de La Raza, Oakland, CA USA; 6 School of Medicine, University of California San Diego, San Diego, CA USA; 7 Department of Medicine, Division of General Internal Medicine, University of California San Francisco, San Francisco, CA USA

**Keywords:** Trauma inquiry, Sexual and reproductive health, Trauma-informed care, Clinician burnout

## Abstract

**Background:**

Trauma impacts sexual and reproductive health. Trauma inquiry is an opportunity for clinicians to identify and respond to trauma histories. The Trauma and Resilience-informed Inquiry for Adversity, Distress, and Strengths (TRIADS) framework could be relevant to sexual and reproductive health clinicians. The objectives of this study were to: (1) explore application of the TRIADS framework to sexual and reproductive health, (2) develop and evaluate a TRIADS-based training for sexual and reproductive health clinicians, and (3) identify practices in trauma inquiry and response in sexual and reproductive health settings.

**Methods:**

A narrative literature review informed the development of trainings (a webinar and collaborative learning groups) on applying TRIADS to sexual and reproductive health. Training participants completed an online survey of implementation outcomes. We used Wilcoxon matched-pairs signed-rank tests to compare self-reported comfort with trauma screening and response before, immediately after, and four months after the training. Collaborative learning groups were recorded, transcribed, and analyzed based on a deductive approach.

**Results:**

Among 117 webinar participants who completed the post-webinar survey, 90% were “likely” or “very likely” to use TRIADS in their clinical practice. Among the 15 collaborative learning group participants, few (13%) reported previous formal training on trauma inquiry or response. Most (80–93%) cared for patients with risk factors for trauma, including substance use, unstable housing, and intimate partner violence. After the collaborative learning group, participants’ comfort with responding to patients’ trauma disclosure, applying the TRIADS framework, and performing a trauma-informed pelvic exam increased. Collaborative learning group participants reported increased resilience and decreased burnout since participation. The following themes emerged from qualitative analysis: Inquiring about Adversity (e.g., building trust before trauma inquiry), Assessing Distress (e.g., practicing trauma-informed pelvic exam), and Identifying Strengths (e.g., promoting reproductive empowerment). Participants reported time and burnout as major barriers to trauma inquiry in sexual and reproductive healthcare.

**Conclusions:**

This study found a meaningful impact of applying the TRIADS framework to sexual and reproductive healthcare. Participation in the webinar and collaborative learning groups demonstrated increased comfort in trauma inquiry and decreased burnout over time. Clinician training in trauma inquiry in sexual and reproductive health settings may benefit both patients and clinicians.

**Supplementary Information:**

The online version contains supplementary material available at 10.1186/s12978-025-02135-6.

## Abstract (plain language summary)

Trauma is an emotional response to a distressing event and can affect health. Certain types of trauma, like sexual violence, can affect sexual and reproductive health especially. Because of this, clinicians who provide sexual and reproductive health care should know how to ask about past trauma and respond to patients in a way that promotes healing. In this study, we developed a training for clinicians on how to talk to patients about trauma in a sensitive way to improve their sexual and reproductive health. We also held discussion groups where clinicians could brainstorm ways to improve the way they provide sexual and reproductive health care to people who may have experienced trauma. Our training was effective in improving clinician comfort with talking to patients about trauma as it relates to their sexual and reproductive health. Clinicians also talked about ways to promote their own wellbeing so that they can best care for their patients. We found that training in trauma inquiry in sexual and reproductive health settings may benefit both patients and clinicians.

**Supplementary Information:**

The online version contains supplementary material available at 10.1186/s12978-025-02135-6.

## Introduction

Trauma, including physical, psychological, and emotional trauma, impacts sexual and reproductive health [1–3]. Adversity and trauma can result from a variety of events and life experiences, such as adverse childhood experiences (ACEs), intimate partner violence (IPV), war, and racism. Trauma is associated with adverse sexual and reproductive health outcomes, including increased rates of sexually transmitted infections, gynecological procedure-related pain, need for emergency contraception, and rates of abortion [4–12]. Trauma also impacts patient experiences with healthcare, including pelvic examination and gynecological procedures [13]. For example, a patient who has experienced sexual violence may find a pelvic examination re-traumatizing.

Despite the strong connection between trauma and sexual and reproductive health, studies show that few clinicians providing sexual and reproductive healthcare routinely ask patients about trauma [14, 15]. Further, there is a lack of guidance around trauma screening and inquiry in sexual and reproductive health settings, such as primary care and obstetrics and gynecology clinics. Although the United States Preventive Services Task Force (USPSTF) provides a Grade B recommendation for routine IPV screening for women of reproductive age [16] and the American College of Obstetrics and Gynecology (ACOG) recommends screening for IPV, sexual assault, and childhood sexual abuse [13], these recommendations are not universally implemented as standard of care and there is variable guidance around how to screen and respond to patients who disclose trauma [17]. To address trauma, experts recommend practicing trauma-informed care (TIC), an approach that realizes the widespread impact of trauma, recognizes the signs and symptoms of trauma, responds to trauma, and resists re-traumatization; TIC is also grounded in six principles: safety, trustworthiness and transparency, peer support, collaboration and mutuality, empowerment, voice and choice, and cultural, historical, and gender issues [18]. TIC seeks to contextualize health with an understanding of trauma’s impact, empowering clinicians with tools to promote patient healing and resilience. However, there is a gap in TIC training in sexual and reproductive health settings [13, 19, 20]. Clinicians providing sexual and reproductive healthcare, such as physicians, midwives, and nurse practitioners, frequently care for patients impacted by trauma but may lack training that could improve patient experiences and reduce clinician burnout [21]. Further, clinicians may feel unprepared for the complex legal aspects of trauma related sexual and reproductive healthcare, such as mandated reporting [22], which can further contribute to burnout.

Trauma screening and inquiry when caring for patients with sexual and reproductive health needs offers an opportunity for clinicians to identify and respond to patients’ experiences with trauma. Clinicians can use TIC practices when addressing sexual and reproductive health needs to promote healing and positive outcomes through frameworks like Trauma and Resilience-informed Inquiry for Adversity, Distress, and Strengths (TRIADS) [23]. TRIADS was initially developed for primary care settings as a follow up to ACEs screening; it emphasizes the importance of an empathetic, respectful, and equity-based TIC approach. The TRIADS framework guides clinicians to discuss trauma with patients through a three-pronged approach: inquiring about adversity, recognizing signs of distress, and identifying patient strengths. The TRIADS framework may be applied to sexual and reproductive healthcare, such as family planning, contraception, abortion, and sexually transmitted infection testing and treatment. Thus, the objectives of this study were to: (1) apply the TRIADS framework to sexual and reproductive health, (2) develop and evaluate a TRIADS-based training for clinicians providing sexual and reproductive healthcare, and (3) identify best practices in trauma inquiry and response in sexual and reproductive health settings.

## Methods

We used a concurrent mixed methods approach [24] for this study exploring the TRIADS framework in sexual and reproductive health settings. Study activities were designated as exempt from the University of California San Francisco and University of California San Diego IRB. Participants provided verbal informed consent to participate. The Consolidated Criteria for Reporting Qualitative Research (COREQ) reporting guideline checklist was completed to ensure quality of qualitative research was upheld [25].

### Participants

Participants in this study included clinicians (e.g., physicians, midwives, nurse practitioners, nurses, and physician assistants) practicing in the state of California who provide any kind of sexual and reproductive healthcare (e.g., STI testing and treatment, abortion care, and contraceptive care).

### Reflexivity statement

The interdisciplinary research team identified as multi-ethnic, Asian, and white, and came from various clinical and academic backgrounds including general internal medicine, family planning, midwifery, emergency medicine, and sociology. These diverse perspectives provided a unique lens for training development, implementation, and data analysis.

### Procedures

#### Training development

To develop a training on trauma inquiry and response in sexual and reproductive health settings, two members of the research team (ZM, MS) conducted a literature review and environmental scan of peer-reviewed literature, clinician trainings and webinars, online resources, and patient educational materials on trauma inquiry and response in sexual and reproductive health settings, using the following PubMed search: ((trauma[Title/Abstract]) OR (intimate partner violence[Title/Abstract]) OR (adverse childhood experiences[Title/Abstract])) AND ((inquiry[Title/Abstract]) OR (screening[Title/Abstract])) AND ((family planning[Title/Abstract]) OR (reproductive health[Title/Abstract])) AND ((English[Language]) AND (“2010/07/31“[Date - Publication] : “2023/07/31“[Date - Publication])) AND United States of America (*N* = 43 articles). Additional resources such as websites, webinars, and patient education materials (*N* = 6) were identified through professional listservs and internet searches. Through regular team meetings, findings were analyzed and mapped to the tripartite TRIADS framework: (1) inquiring about patient adversity, (2) recognizing signs of patient distress, and (2) identifying patient strengths. We summarized best practices within each category, which informed training development.

#### Initial webinar and survey

Based on the training developed above, our team led one 90-minute, live webinar through the California Office of Family Planning in December 2023. Participants were recruited through the Family Planning, Access, Care, and Treatment (Family PACT) program, which includes nearly 2,300 clinicians serving Medi-Cal patients. The first sixty minutes of the webinar included background information on trauma and resilience, followed by a clinical case that applied the TRIADS framework to provision of sexual and reproductive healthcare. Thirty minutes were dedicated for a question and answer session. The webinar was recorded and made available on the Family PACT website [26]. A post-training survey (Supplement 1) was administered online to evaluate implementation outcomes.

#### Collaborative learning group participant recruitment

As a follow up to the initial webinar, we held collaborative learning groups (CLGs) for in-depth discussion of trauma inquiry in sexual and reproductive health. Clinicians from a variety of clinical backgrounds (physicians, advanced practice providers, certified nurse midwives, and nurses) were recruited to participate in a live, virtual, and discussion-based training on trauma inquiry and response utilizing the TRIADS framework. Participants were recruited via convenience sampling from the initial webinar as well as professional listservs from author institutions. Recruitment emails included five possible dates to participate in collaborative learning groups during 12 − 1:30 pm, and participants were asked to mark their availability for all dates, noting a maximum of 10 participants in each date to foster small-group, collaborative discussion. Participants were also asked about eligibility in the recruitment emails; they were eligible if they provided sexual and reproductive healthcare in the state of California and were given $50 for their participation.

#### Collaborative learning group content and structure

The content and structure of the CLGs were developed based on results and feedback from the initial webinar. The research team met with the TRIADS developers to review the questions that came up in the initial webinar Q&A session as a way to understand participant training needs. For example, participants asked about specific clinical tips, such as how to perform a trauma-informed pelvic exam, as well as legal considerations, such as mandated reporting requirements. Further, after scheduling their session, CLG participants were given a pre-survey (Supplement 2) asking about training needs and specific sexual and reproductive health cases they wanted to discuss. The research team meet biweekly to ensure findings from the initial webinar were translated to CLGs in a way that met participants’ training needs. A total of five CLGs were scheduled and held between March – June 2025. CLGs were 90-minute live, virtual, discussion- and case-based trainings utilizing the TRIADS framework. CLG facilitators included members of the research team (MS, SJ, KL), as well as TRIADS expert consultants (AL, KS). A slide deck with facilitator notes was used to guide CLGs (Supplement 3). MS was the primary facilitator, and all CLGs were facilitated by MS, along with either SJ or KL and AL or KS, to ensure that there was at least one clinician facilitator and one TRIADS expert consultant. MS started each CLG with a grounding exercise followed by background information on trauma and sexual and reproductive health. MS then described the TRIADS framework and provided an example of how to apply it to SRH. This 15-minute didactic portion ended with information on clinician wellbeing and secondary trauma. After, participants introduced themselves and provided case examples for a facilitated discussion on effective practices for trauma inquiry and response in sexual and reproductive health settings.

### Measures and data analysis

#### Online survey quantitative analysis

Initial webinar participants completed an online survey after the webinar (Supplement 1) to evaluate implementation outcomes, which were analyzed using summary statistics. CLG participants completed an online survey (Supplement 2) assessing demographics and self-reported comfort in practicing TIC using a 5-point Likert scale related to the training content before, immediately after, and four months after the training. The survey administered four months after the CLG also assessed changes in clinical practice, self-reported resilience, and self-reported burnout. We used descriptive statistics to describe participant demographic characteristics and the Wilcoxon matched-pairs signed-rank test to compare self-reported comfort with trauma screening and response before, immediately after, and four months after the training, with statistical significance defined at *p* < 0.05. All analyses were conducted using StataSE version 18.5.

#### Collaborative learning groups qualitative analysis

CLGs were conducted, recorded, and transcribed using Zoom software [27]. Only the discussion portion (average of 60 min) of the CLGs was audio recorded and transcribed, and all five transcripts were de-identified and corrected for discrepancies by the research team (EN, MS). Qualitative data included both participants and facilitators, where we analyzed individual data, group data, and group interaction data [28]. A codebook was developed deductively based on the TRIADS framework (Supplement 4). Thematic saturation was reached after conducting 5 CLGs. A deductive qualitative analysis followed an iterative process in three phases: (1) organizing and sorting the data, (2) understanding and interpreting the data, and (3) explaining the data [29]. Three researchers (MS, MA, EN) independently conducted blind open coding to first organize and sort the data using Dedoose software [30], guided by thematic categories inspired by the TRIADS framework. Regular meetings were held to discuss the blind open coding process, resolve discrepancies, and collaboratively refine the categories to understand and interpret the data, limiting bias and enhancing analytic rigor. After achieving consensus through regular meetings, this was followed by the third phase to explain the data (MS, MA) and synthesize the data into cohesive themes through individual analysis followed by biweekly meetings.

## Results

### Webinar

California-based clinicians and staff registered (*N* = 605) and attended (*N* = 298) the initial webinar, among whom 39.3% (*N* = 117) completed post-webinar surveys. Table 1 shows participant demographic characteristics; most respondents identified as women (88.0%); 41.4% were white, 23.4% identified as Latine, and more than half were nurses (63.7%). Table 2 reports webinar participant implementation factors related to applying the TRIADS framework when providing sexual and reproductive health care; most (90.6%) respondents reported they were “likely” or “very likely” to use TRIADS in clinical practice. Barriers to using TRIADS in clinical practice included lack of time, training, and referral resources. Facilitators included confidence in asking and responding to patients with trauma. Nearly half of the respondents were interested in attending a follow-up collaborative learning group.

**Table 1:** Characteristics of clinicians who attended a webinar on trauma inquiry and response in sexual and reproductive health

Characteristic	Summary *N* = 117* (%)
Gender	
Woman	103 (88.0)
Man	10 (8.6)
Non-binary/Genderqueer	2 (1.7)
Prefer not to say	2 (1.7)
Race & Ethnicity	
White or European origin	48 (41.4)
Hispanic, Latino, or Spanish origin	27 (23.3)
East, Southeast, or South Asia	15 (12.9)
Black or African American	10 (8.6)
Multiple	7 (6.0)
Other	4 (3.5)
Native American or Alaska Native	2 (1.7)
Native Hawaiian or Pacific Islander	2 (1.7)
Middle Eastern or North Africa	1 (0.9)
Clinical Training	
RN	38 (34.6)
NP	28 (25.5)
Other	24 (21.8)
MD/DO	7 (6.4)
MA	7 (6.4)
CNM	4 (3.6)
PA	2 (1.8)
Years in Practice	
1–5	32 (29.1)
6–10	12 (10.9)
11–20	34 (30.9)
21–30	22 (20.0)
30+	10 (10.1)

*Responses may not to add to total *N* as participants were not required to answer all questions

**Table 2:** Implementation factors related to applying the TRIADS framework when providing sexual and reproductive health care

Implementation Question	*N* = 117 (%)
How likely are you to use the TRIADS framework in your family planning clinical practice?	
Very Unlikely	7 (6.0)
Unlikely	4 (3.4)
Likely	70 (59.8)
Very likely	36 (30.8)
What are some of the reasons that it might be easy to use the TRIADS framework in your family planning clinical practice? (select all that apply)	
Confidence in how to respond to patient trauma	54 (46.2)
Comfort with using a small amount of time during visits to discuss trauma	53 (45.3)
Comfort with asking patients about trauma	52 (44.4)
Adequate training	44 (37.6)
Adequate referral resources	36 (30.8)
Other	4 (3.4)
What are some reasons that it might be difficult to use the TRIADS framework in your family planning clinical practice? (select all that apply)	
Time constraints	57 (48.7)
Lack of training	41 (35.0)
Lack of referral resources	36 (30.8)
Unsure how to respond to patient trauma	26 (22.2)
Discomfort with asking patients about trauma	16 (13.7)
Other	13 (11.1)
Are you interested in participating in a follow-up training that will entail a 1-hour discussion with other family planning clinicians?	
Yes	52 (45.6)
No	62 (54.4)

### Collaborative learning groups

#### Quantitative results

**Fig. 1:**
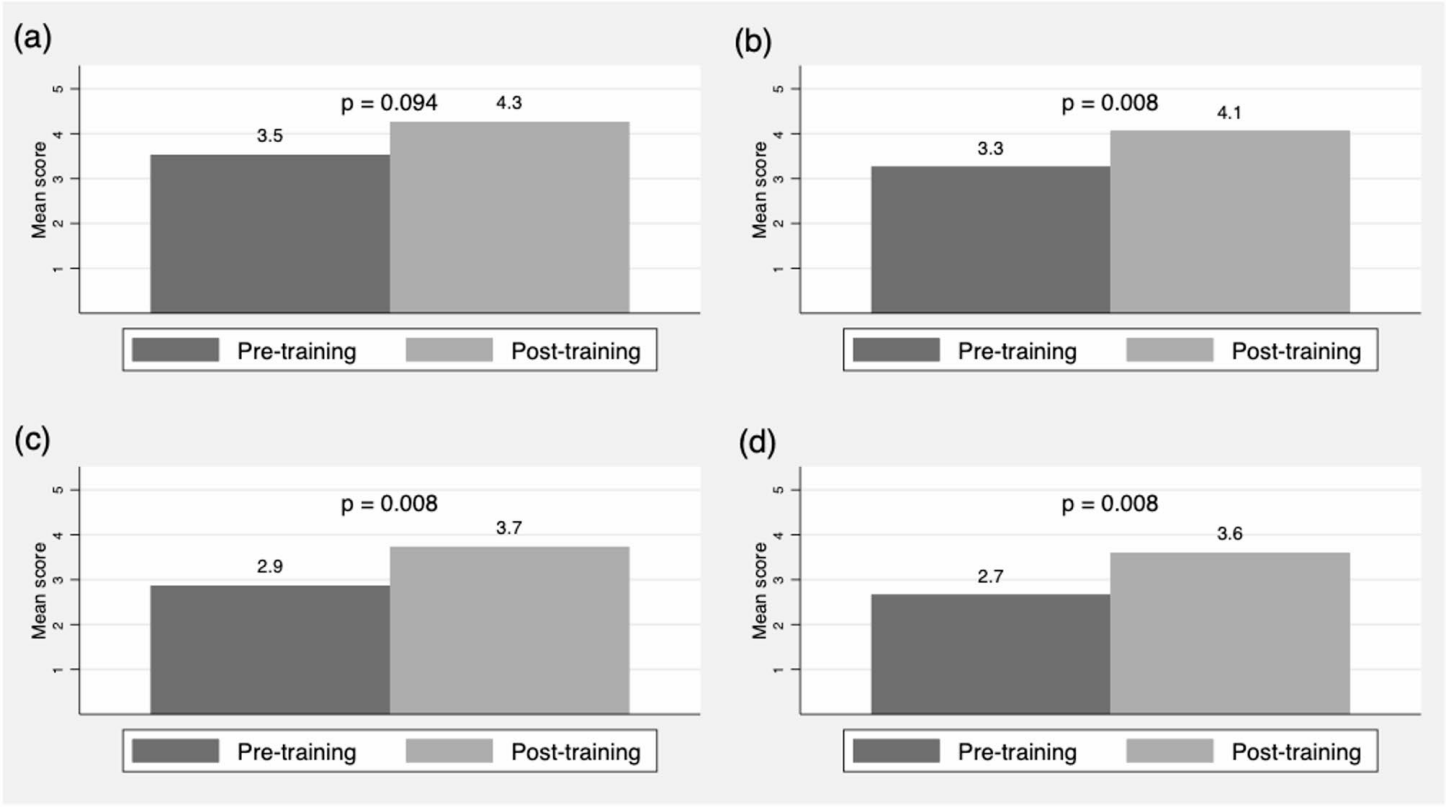
Self-Reported Comfort in Trauma-Informed Care Before and After Participation in a Collaborative Learning Group on Trauma Inquiry in Sexual and Reproductive Health. This figure shows collaborative learning group participant self-reported comfort with (**a**) asking patients about past trauma, (**b**) responding to patient’s disclosure of trauma, (**c**) performing a trauma-informed pelvic exam, and (**d**) applying TRIADS framework in clinical practice. The dark gray bar refers to comfort before participation (pre-training) and the light gray bar refers to comfort after participation (post-training), with associated *p*-values obtained from the Wilcoxon signed-rank test. Comfort was reported on a 5-point Likert scale, where 1 = very uncomfortable, 2 = uncomfortable, 3 = neither comfortable nor uncomfortable, 4 = comfortable, and 5 = very comfortable

Table 3 describes the CLG participant demographic and clinical training characteristics, as well as baseline clinical experiences related to trauma. Of the 15 total participants (average of three per CLG), all self-identified as women. Participants came from various clinical backgrounds including MD/DO (40%) and NP/RN/CNM (46.8%) and worked at various clinical settings including academic clinics (33%) and family planning clinics (26.7%). Few reported formal trauma-related training through work (13%) or school (20%). Most cared for patients with risk factors for trauma, including substance use (87%), unstable housing (80%), and past IPV (93%); 40% reported caring for patients with a history of trauma at least once per week. After the training, self-reported comfort with responding to patients’ trauma disclosure increased (*p* = 0.008), as did comfort with applying the TRIADS framework in clinical practice (*p* = 0.008) and performing a trauma-informed pelvic exam (*p* = 0.008) (Fig. 1, Self-Reported Comfort in Trauma-Informed Care Before and After Participation in a Collaborative Learning Group on Trauma Inquiry in Sexual and Reproductive Health). Comfort with asking patients about past trauma was high at baseline but still increased immediately after the training (*p* = 0.094). Among the nine participants who completed the 4-month survey, comfort with responding to patients’ trauma disclosure was higher four months after compared to before training (*p* = 0.031). These participants shared TIC practices they have continued to make longitudinally since CLG participation, such as focusing on patient strengths and peer support resources. Additionally at 4-month follow-up, 44.4% reported increased resilience and 55.6% reported decreased burnout since participating in the training.

**Table 3:** Characteristics of clinicians in a collaborative learning group on trauma inquiry in sexual and reproductive health settings

	Summary *N* = 15 (%)
Gender	
Female	15 (100.0)
Race & Ethnicity	
Black or African American	2 (13.3)
East, Southeast, or South Asia	1 (6.7)
Hispanic, Latino, or Spanish origin	3 (20.0)
Middle Eastern or North Africa	1 (6.7)
White or European origin	7 (46.7)
Multiple	1 (6.7)
Clinical Training	
MD/DO	6 (40.0)
PA	1 (6.7)
NP	4 (26.7)
CNM	1 (6.7)
RN	1 (6.7)
Other	1 (6.7)
Prefer not to answer	1 (6.7)
Specialty	
Family Medicine	4 (26.7)
Obstetrics and Gynecology	3 (20.0)
Preventive Medicine	1 (6.7)
Midwifery	1 (6.7)
Other	6 (40.0)
Years in Practice	
1–5	7 (46.7)
6–10	1 (6.7)
11–20	3 (20.0)
21–30	4 (26.7)
Type of Healthcare Facility	
Federally qualified health center	3 (20.0)
Community-based clinic	2 (13.3)
Academic-affiliated clinic	5 (33.3)
Refugee health clinic	2 (13.3)
Family Planning Clinic	4 (26.7)
Other**	3 (20.0)
How often do you work with patients who have disclosed a history of trauma?	
Rarely	1 (6.7)
At least once per month	6 (40.0)
At least once per week	6 (40.0)
Daily	2 (12.5)
How have you become aware of trauma your patients have experienced? (Select all that apply)	
Patient chart review	9 (60.0)
Screening tool	2 (13.3)
Patient’s direct disclosure	13 (86.7)
Patient’s response to direct inquiry	14 (93.3)
Clinical suspicion based on history and/or exam	8 (53.3)
Other	2 (13.3)
Approximately what proportion of the patients you see identify as cis-female?	
None	0
Less than half	1 (6.7)
About half	2 (13.3)
Majority	12 (80.0)
Approximately what proportion of the patients you see identify as transgender and/or gender-diverse?	
None	1 (6.7)
Less than half	14 (93.3)
About half	0
Majority	0
Do you provide healthcare for any of the following populations? (Select all that apply)	
People with substance use disorders	13 (86.7)
People with unstable housing	12 (80.0)
People with a past or current history of intimate partner violence	14 (93.3)
People with a trauma related to being an immigrant, refugee, or asylum-seeker	10 (66.7)
People with a known history of trauma of any kind	11 (73.3)
Other	2 (13.3)
What training around trauma have you received in the past? (Select all that apply)	
No training	2 (13.3)
Formal class/workshop training in school	3 (20.0)
Training class/workshop offered through work	2 (13.3)
Informal training through webinar, articles, or other online resources	13 (86.6)
Other	4 (26.67%)
Have you received training regarding how to perform a trauma-informed pelvic exam?	
No	9 (60.0)
Yes	6 (40.0)

**“Other”: non-profit organization; free standing birth center; partnerships with healthcare and social assistance industry, government agencies and academic institutions

#### Qualitative results

Through facilitated discussion, CLG training participants (T#) and facilitators (F#) from the research team applied the TRIADS framework to patients they took care of with sexual and reproductive health needs. Participants shared clinical cases that were used for discussion involving patients with various trauma histories (e.g., IPV, reproductive coercion, housing instability, ACEs, sexual assault, war-related trauma) and sexual and reproductive health needs (e.g., contraception, STI testing, abortion). Analysis of the CLG discussion resulted in four themes related to how participants applied the TRIADS framework to sexual and reproductive health: (1) Adversity: Inquiring and Responding, (2) Distress: Recognizing and Addressing, (3) Strengths: Identifying and Empowering, and (4) Implementation Factors. Participants repeatedly shared how they practice the three components of TRIADS, but in a non-linear manner (e.g., they may start by recognizing signs of distress rather than starting with inquiry). Further, participants highlighted the interpersonal nature of conversations about trauma and how to navigate the transactional parts (e.g., logistical requirements) with the relational parts (e.g., compassionate communication).

#### Adversity: inquiring and responding

CLG participants noted several approaches to inquiring about experiences with adversity and trauma (*n* = 276 excerpts coded for “Adversity”). Table 4 lists several key phrases and verbal approaches participants use to ask about trauma for patients seeking sexual and reproductive healthcare. Some participants noted they include a routine screening question in all their patient intake forms that specifically asks about sexual trauma or IPV. These participants followed up any positive responses with dialogue with the patient. Participants that practiced trauma inquiry, either as a follow up to screening or inquiry by itself, noted that building trust with the patient before discussing trauma is crucial: “Establishing a level of trust that lends itself to more comprehensive and customized care” (T12). Participants who saw patients for sexual and reproductive health concerns in primary care settings shared that continuity of care was a key factor in building trust over time, while others who worked in acute care settings (e.g., asylum seeker shelter, family planning clinic) noted the difficulty of trust building in one-time encounters. Regardless of setting, participants shared that normalizing trauma inquiry is an important initial step, followed by personalization to each patient:

*Normalizing it is saying*,* “This is part of what we ask everyone.” And then the subjectivity part is interesting too. It’s not somebody else’s definition of what’s traumatic*,* it’s something that [the patient] found overwhelming. I think every patient requires a different approach*,* based on what their background is*,* what language they use. We wanna reflect that back to them (F4).*

**Table 4:** Key phrases for trauma inquiry and response in sexual and reproductive health settings

Inquiring about Adversity
*Can you tell me more about what happened? For example*,* was there anything scary or unwanted when you had sex?*
*Violence is an unfortunately common experience for people. Was this a part of your experience as well?*
*Was the sex you had something you wanted to do?*
Responding to Patient Disclosure of Trauma
*I’m so sorry this happened to you.*
*I want you to know that this is not your fault.*

Participants noted that in sexual and reproductive health settings, where the patient may present with their partners, it was critical to inquire about trauma in private, one-on-one, and, if needed, with the help of a medical interpreter who was not a family member. This is especially relevant when asking about IPV. However, participants shared that when discussing trauma, sexual and reproductive health decision-making such as contraceptive choices often come up. During these conversations, patients often bring up their partners: “I’ll notice people bringing up their partners a lot in the conversation, in ways that seem kind of interesting when, you know, you’re talking to a patient about what they want to do with their own body. So, I think having an open conversation with patients in that regard can be a really helpful way to get clued into [trauma].” (F5). One participant shared uncertainty around how to involve partners in the context of trauma: “If I’m worried about coercion of contraception, do I try and engage the partner to further understand? Or do I just try and remain in direct alliance with my patient? I don’t know.” (T2). Another participant shared an approach navigating these dynamics, noting that there is a strong gender norm in certain cultures where some women may feel more comfortable including their partners in conversations about contraception, but others may want to learn about contraceptive methods that can be hidden from their partners:

*I’m working with a pretty big population of Guatemalan ladies and the cultural component here is big*,* especially when*,* in their culture*,* the man makes the decisions and they obey*,* and that’s pretty much what it is. So it’s*,* it is very challenging to tell them that they can choose*,* and it’s their bodies and their choice*,* and I’ve had*,* actually*,* two patients that they came to take the IUD out because the partner wanted another baby*,* and they already have 5 babies and they’re in their early thirties and so*,* I always have consultations when patients want a removal of a LARC before I have the actual appointment for the removal*,* just for that reason*,* just to discuss why they want to take it out*,* and what’s their plan? And then they’re pretty open about it*,* and it’s not*,* that they’re like*,* yes*,* my husband wants another baby*,* and then when I ask them*,* ‘what do you want?’*,* and it’s like a shock for them*,* like*,* what do you mean*,* what do I want? So*,* that’s one thing. What I offer is*,* I do have discussions with them about other options. For example*,* we do the DEPO-shot so that there’s no way of the husband to know if she’s getting it or not. I feel like the cultural part is it’s a big challenge especially with the population that I’m working with (T14).*

With cultural contexts in mind, participants brought up intentionally involving the partner: “I try to encourage the patients to bring their partners with them. I feel like discussing with both of them at the same time gives me more perspective of what’s happening. And I feel like education is the big piece that is probably missing with the population that is from a different country or from a different culture.” (T14). One participant shared an approach to inquiry regarding sexual and reproductive health decision-making between partners:

*My next question would be to turn to the partner and speak to both of them together. I say*,* “This is such an intimate a decision*,* isn’t it? How do the two of you come to that decision? Are you both on board with that? Is this something that was easy to come about? Or did it take a lot of negotiation? And do you both feel equally convinced that this is the right decision for both of you?” And so even the way of posing that normalizes. And I might say*,* for example*,* “Sometimes couples really are on the same page*,* and sometimes they really have different points of view about it. What is it like for the two of you?” (F3).*

Participants noted that inquiring about trauma requires being prepared with responding to patient disclosure of trauma and having resources available. Participants shared various approaches to responding to trauma disclosure, such as validating patient experiences, providing empathetic support, and offering the patient resources if appropriate.

Participants shared that inquiring about trauma in sexual and reproductive health settings may bring up important legal considerations. For example, clinicians discussed mandated reporting requirements related to IPV or sexual assault:

*So if you ask [about trauma]*,* and then they say yes*,* that they did experience the trauma…the reporting piece - being a mandated reporter. I wonder if you disclose anything about that beforehand*,* or*,* I don’t know*,* cause it - it could lead into a different type of situation (T6).*

In response, participants shared key approaches to disclosing mandated reporting requirements as well as offering alternative sources of support:

*I always try to - if you see it coming*,* and you can’t always - is to let them know that I’m a mandated reporter. I’m happy to listen. I’m here. I want to support them*,* but that we have mental health providers that are not mandated to report that. So*,* if they want to share that in a safer*,* you know*,* environment*,* where they don’t - they aren’t forced to make that decision about reporting*,* that*,* you know*,* I’m happy to connect them and still provide care for them (T4).*

In this way, participants stated that a key trauma-informed approach is to let patients know of their mandated reporting requirements upfront before asking about trauma, which offers the patient more agency to disclose only what they feel comfortable sharing. However, participants also noted that sometimes this is not possible due to clinic workflows:

*[Screening] questions get asked before the provider ever sees the patient routinely. I mean*,* it’s completely routine for every single visit. So*,* it is a challenge*,* cause that’s really different for me from where I came from before*,* where that was definitely something that the that the clinician*,* you know*,* the physician or NP or PA would have asked*,* and it wouldn’t have been*,* those questions wouldn’t have been asked by the medical assistant. And though there’s very empathic*,* skilled medical assistants*,* I just know that they just don’t get that much training on how to respond to any of these answers other than*,* ‘okay*,* I’m gonna tell your provider’*,* you know*,* ‘I’m gonna let them know about what we discussed’ and that*,* you know*,* I think*,* can be very*,* very anxiety*,* provoking and triggering - just that whole interaction (T11).*

Further, participants noted that unique, intersectional medical and legal considerations may arise for patients who are presenting for sexual and reproductive healthcare after a sexual assault. For example, participants working in a college health setting shared Title IX requirements.

#### Distress: recognizing and addressing

Participants shared various ways to recognize signs of distress (*n* = 248 excerpts coded for “Distress”) in individuals seeking sexual and reproductive healthcare, noting that patients may exhibit signs of distress through behavior, language, or reactions to physical exams, particularly pelvic exams.

For some it was straightforward to recognize signs of distress: “It was very obvious, as soon as she took her hat off, it was like there were bruises all over, so it was very obvious, and marks on her neck.” (T16). Other participants shared various ways of asking about signs of distress when taking a medical history, noticing “…how the body is responding. Do you find yourself not sleeping? Or how is your sleep? You might say, or what about your appetite? What about your energy level? Are there ways that you want to live your everyday life that you find that you’re just not able to?” (F3). Participants also shared times when it was difficult to recognize signs of distress and used the TRIADS framework in a non-linear manner, connecting back to inquiring about adversity or identifying patient strengths.

Participants shared approaches to recognize and address signs of distress through a trauma-informed pelvic exam. Participants recommended establishing rapport before the exam, noting that this may require a separate visit. Participants suggested inviting the patient to identify measures that may make them more comfortable, such as using a support person (e.g., family member or doula), listening to music, or using a heating pad. Further, clinicians can offer gender-concordant chaperones and examiners when possible, discuss pain control options before a gynecological procedure, and take ample time with obtaining consent. Participants highlighted the importance of asking for permission at every step of the exam and ensuring the patients know that they are in control of the exam and can stop at any time. Participants also offer patients self-swab options for STI testing or patient self-placement of speculum and/or vaginal ultrasound probe when needed. Table 5 shows words to avoid and words to use instead to prevent re-traumatization. Participants noted that it is important to avoid “bedroom” language, as these words may have been spoken to patients during a traumatizing encounter, and to use patient-friendly, clinical language instead.

**Table 5:** Recommended Language with Trauma-Informed pelvic exams

Words to Avoid	Words to Use Instead
*Bed*	*Table*
*Spread your legs*	*Let your knees fall to the sides*
*Hand*,* finger*	*Glove*
*Stirrups*	*Footrests*
*Relax*	*Gently allow your hips to fall towards the table*

#### Strengths: identifying and empowering

Participants noted various ways to identify patient strengths, empower patients, and provide resources to address sexual and reproductive health and trauma-related concerns (*n* = 216 excerpts coded for “Strengths”). Many participants commented on the strength of a patient showing up to the clinic. Participants also noted that they ask about patient support persons, such as family, friends, peer support specialists, abortion doulas, or others. For example, a participant shared a story of a patient experiencing IPV whose partner provided her stable housing:

*So I could make an observation or reflection like*,* ‘Having known you for this amount of time*,* I’m so impressed by all the things that you’ve done to keep yourself healthy and safe through this pregnancy.’ That would include everything that she did. It sort of includes being with him*,* right? And finding a partner that she thinks is stable and being housed. And just*,* like*,* it’s a big lift for her to be doing all this stuff. And just honoring that desire to be healthy and safe. What does she think would be the best thing*,* would she want birth control? Is there a way that I could partner with her*,* either by having a conversation with her husband*,* or by talking to her about options that don’t have anything to do with him*,* like invisible options*,* that could help her continue in that vein*,* of doing the safest thing for her own health and for the baby too*,* I think that’s another really good one to bring in*,* just because people are so motivated*,* especially people who experience trauma*,* are so motivated*,* more so for their child’s health than their own*,* but I find sometimes*,* when I’m like stuck between*,* should I talk about more of the adversity? Should I explain the link to health*,* just noticing a strength and reflecting it back*,* and kind of like*,* give them a little rush of energy to say to locate their current desire in a thread that’s really about self-respect and safety (T2).*

Further, participants discussed ways to empower patients with their choices around contraception by offering various options that consider a trauma history. As above, participants highlighted the utility of “invisible” contraception for patients who are experiencing reproductive coercion. For some participants, this worked well: “And so, we ended up putting it in and not telling her partner she had one. He never noticed, and she still is not pregnant” (T2). However, other participants noted that discussing this also requires a discussion around how the patient may need to address their partner that suspects “invisible” contraceptive use if sex is not resulting in pregnancy.

Participants practiced cultural humility and highlighted cultural practices as strengths and to start the conversation around healthy sexual and reproductive care. For example, one participant suggested the following for a patient who had several prior pregnancies with elevated health risk:

*There’s something I want to talk to you about*,* which is*,* you have a wonderful big family*,* and I know how big families are celebrated in your culture*,* and it’s a wonderful thing. And*,* at the same time*,* studies have told us that having very closely spaced pregnancies can really be a health risk. Do you have health concerns that you are worried about? (F3).*

Finally, as an approach to identifying patient strengths, participants shared several resources for patients with a history of trauma, including hotlines (e.g., National Domestic Violence Hotline) and mental health resources (e.g., primary care mental health integration). Other resources included anonymous STI notification (e.g., tellyourpartner.org) and safety planning (e.g., myPlan). Participants took a universal education approach, providing patients with trauma related resources regardless of disclosed trauma (e.g., providing all patients with National Domestic Violence Hotline). In college health settings, participants discussed Title IX resources.

#### Implementation factors

Participants shared various implementation factors when applying the TRIADS framework to sexual and reproductive clinical care (*n* = 133 excerpts coded for “TRIADS Barriers” and *n* = 285 excerpts coded for “TRIADS Facilitators”). Time was a major barrier: “In a 15 min, I don’t feel like I always have the ability to get that full picture. I try and ask, who’s your support person, who’s helping you with taking care of yourself while you take care of a kid, but no, I don’t think I can [ask about trauma] in a 15 minute visit” (T2). In addition to time constraints, another major barrier was the lack of trauma related resources available in clinics and health systems: “I think that it’s hard when I don’t know the resources. That’s the part that feels hard in the framework.” (T2).

Participants also shared ethical barriers to providing trauma-related sexual and reproductive healthcare such as moral conflict regarding pregnancy options counseling after a sexual assault. One participant shared an experience where the patient asked for her opinion:

*A lot of our patients who come in with history of trauma ask*,* ‘What should I do?’ or ‘Just tell me what to do.’ And for this patient*,* she had history of trauma - physical abuse in the past*,* sexual abuse*,* substance-use disorder and…had this unplanned pregnancy…and it was also a very complex situation*,* … asking*,* ‘What should I do? What do you think I should do?’ And I think it’s always very complicated approach (T19).*

In response, a participant shared approaches to addressing this:

*When somebody tells us*,* ‘what do you think I should do?’ The first thing*,* is to acknowledge what they did. That*,* and how honored we feel*,* that you want our opinion. Thank you for asking for my opinion. It gives me a sense of*,* that you value what I might have to say*,* and at the same time*,* I want to acknowledge that… I will not need to live with whatever consequences there are*,* and that*,* and that you have the wisdom to think*,* ‘if I did this*,* what would it be like for me? And if I did this [other choice]*,* what would it be like for me?’. Can we take that time to think together? … So*,* this is really important - legitimizing what important things you are considering. And I think*,* what was already said*,* that these are not decisions that are made in one session (F3).*

As above, participants again highlighted the importance of time and multiple visits to work through these ethical considerations with patients.

Clinician emotional response to patient disclosure of trauma was another important implementation consideration. Several participants felt emotional discussing patient cases: “Even thinking about right now, it’s like making a little emotional. I just feel so much for her. I didn’t realize I’d get emotional. Sorry. I guess that’s part of the whole deal” (T12). Participants repeatedly shared their appreciation for the small group discussion and support as a way to promote their wellbeing as clinicians. One participant responded to another participant getting emotional: ”I appreciate you. I appreciate you, and my eyeball sweat a lot, too. So, it’s, it’s healthy. That’s how we process. Right? You have to process” (T12). Further, in the four-month post-CLG survey, participants shared continued resilience practices, such as setting boundaries, taking time off, and embracing discomfort. Participants appreciated each other’s support in the conversation, as well as having the space and time to process together with peers. In the immediate post-CLG survey, one participant shared: “I really love this collaborative group experience. It gave us practical ways to provide trauma informed care. I learned so much from this group and very grateful to be a part of this. The small group setting and real clinical scenarios were extremely helpful. Thank you so much for this opportunity (T4).” In this way, peer support was a facilitator to applying TRIADS to sexual and reproductive healthcare, where participants normalized and processed feelings with each other.

## Discussion

This study explored approaches to trauma inquiry and response in sexual and reproductive health contexts using the TRIADS framework. Our results show that while clinicians provide sexual and reproductive healthcare for a patient population that is at high risk for trauma, few had formal TIC training. Clinician TIC comfort increased after participation in a TRIADS-based CLG. Through the CLGs, we found that trauma inquiry and response in sexual and reproductive health settings presents unique challenges and opportunities, including building trust in brief encounters, focused trauma inquiry, legal and ethical factors, and clinician wellbeing and burnout prevention.

### Building trust in brief encounters

Many sexual and reproductive health encounters are one-time or urgent care related, such as abortion care or STI treatment. In line with the literature, our study participants emphasized the importance of building a trusting relationship before inquiring about trauma [15, 31–33]. Given this, clinicians providing sexual and reproductive healthcare face the challenge of quickly establishing rapport and trust with patients to support trauma inquiry and appropriate clinical care. Participants shared strategies for building trust in these brief encounters that align with prior studies, including prioritizing patient privacy and confidentiality, using trauma-informed language and non-judgmental communication, explaining procedures and obtaining ongoing consent, offering choices and control over aspects of the visit, and recognizing and validating patient experiences and emotions [18, 34]. Implementing these trauma-informed approaches with a focus on building trust can support clinicians to create a safe environment for patients to share relevant trauma history and foster a therapeutic relationship, even within the constraints of a one-time clinical encounter [35, 36].

Participants in this study highlighted practicing cultural humility [37] as an approach to building trust with patients seeking sexual and reproductive healthcare. Practicing cultural humility allowed clinicians to build trust while also promoting autonomy and empowerment in a truly patient-centered manner, avoiding the pitfalls of a one-size-fits-all approach. For example, our study suggests that promoting autonomy and empowerment may include involving partners in sexual and reproductive health conversations if culturally appropriate; this differs from previous studies that promote a focus on the individual patient alone for sexual and reproductive health decision making [38]. Building trust through cultural humility is also an example of a strengths-based approach to care, where clinicians partner with patients to identify strengths to promote resilience in the context of trauma [18]. Partnering with patients to create a supportive structure for the visit from the start highlights the non-linear nature of applying TRIADS to sexual and reproductive health; each of the three elements of the framework is connected to and supports the others, so clinicians may have flexibility within an encounter for how and when they identify strengths, address adversity and assess for signs of distress. Regardless, building trust, practicing cultural humility, and focusing on strengths are foundational to applying TRIADS to sexual and reproductive health settings.

Further, participants noted that while the kind of continuity of care seen in primary care can be particularly helpful in building trust and can serve as a protective factor [39], clinicians can focus on promoting continuity within the healthcare system at large. Focusing on healthcare access for sexual and reproductive health patients experiencing trauma may help promote patient trust and a feeling of non-abandonment that is also critical in other healthcare settings such as palliative care [40]. Literature shows that trauma-informed systems and clinics can promote patient trust in healthcare as a whole, which may be a critical aspect to trauma inquiry in a single visit [41].

### Focused inquiry on traumas related to sexual and reproductive health

There are several forms of trauma that may affect individuals seeking sexual and reproductive health services, including IPV, ACEs, racism, war violence, and more. While patients may have diverse trauma histories, participants from our study discussed cases where they prioritized screening for traumas that directly impacted sexual and reproductive health, which aligns with due to their high prevalence in those seeking related care [42–44]. This may include IPV, sexual assault, childhood sexual abuse, sex trafficking, reproductive coercion, and other forms of gender-based violence like female genital cutting. These forms of trauma can influence contraceptive choices, pregnancy intentions, STI testing and treatment, and overall reproductive autonomy [2, 3, 45–47]. Participants in our study emphasized the option of “invisible” contraception to promote reproductive autonomy in the setting of reproductive coercion, providing clear clinical recommendations for various situations such as using a copper IUD with the strings cut off pre-insertion if a partner is tracking menstruation [46, 48, 49]. There is evidence that other forms of trauma are related to sexual and reproductive health; for example, individuals with an elevated ACEs score have higher rates of abortion [50–52]. However, clinicians in our study noted significant restrictions with time, and thus, in time-limited settings, clinicians may consider prioritizing trauma inquiry that directly impacts sexual and reproductive health decision-making.

Clinicians providing sexual and reproductive health services should be trained to recognize signs of related traumas and offer universal trauma-informed counseling and patient-centered interventions. Clinic screening tools and protocols should be designed with the specific patient population in mind (e.g., screening for female genital cutting in a refugee clinic or screening for sexual assault when providing abortion care). Further, clinicians can inquire about recent experiences of sexual and reproductive health related trauma with sensitivity and cultural humility, while also assessing for immediate safety concerns. The integration of brief interventions, such as safety planning and referrals to support services, is crucial in these time-limited sexual and reproductive health encounters [41].

At the same time, when continuity of care is possible, such as primary care settings, it may be appropriate and relevant to inquire about diverse forms of trauma. Participants in our study routinely screened for IPV using validated tools, such as HITS, HARK, and/or AAS [53]. There may be a role for additional trauma-related screenings in primary care, particularly as it relates to a patient’s health trajectory over time. This may include the Adverse Childhood Experience Questionnaire for Adults, for example, as there is evidence that ACEs affect adult health in a long-term manner [54].

### Legal requirements and ethical considerations

Clinicians providing sexual and reproductive healthcare navigate complex legal and ethical considerations when inquiring about and responding to trauma. This includes mandatory reporting requirements for certain types of traumas, such as IPV, which vary by state [22]. In our study, participants raised questions about uncertainty around legal requirements and policies related to sexual and reproductive health, such as mandated reporting for IPV and Title IX considerations for sexual assault [55]. Our study shows that clinics and hospital systems should have clear protocols and clinical workflows that involve all staff (front desk, medical assistant, clinician, mental health provider) in place to guide clinicians in fulfilling their legal obligations while maintaining patient trust and autonomy.

Additionally, our study participants shared clinical scenarios that highlight intersectional medical and legal considerations, such as Title IX. While our study included clinicians practicing in California, there are other legal considerations for those practicing in other states. For example, legal requirements in other states may include physician documentation of sexual assault for coverage of abortion services and waiting 72 h before providing abortion care [56]. Further, if a patient receives an abortion after a sexual assault and is pressing charges, clinicians may recommend a procedural abortion to collect DNA evidence [57]. To effectively and compassionately carry out this type of medical-legal clinical decision making, our study suggests that clinicians may benefit from being well-versed in their legal responsibilities as well as the legal rights of survivors, including options for forensic evidence collection, legal advocacy services, emergency contraception, and abortion policy. Offering comprehensive information about these rights in a compassionate manner can empower patients to make informed decisions about their sexual and reproductive healthcare.

#### Clinician wellbeing and burnout prevention

Implementing TIC practices in sexual and reproductive health settings has the potential to improve clinician wellbeing and decrease burnout by providing a context where clinicians can better meet patients’ needs and enhance the effectiveness of care. Providing trauma related sexual and reproductive healthcare can also be emotionally demanding for clinicians, potentially contributing to burnout and secondary traumatic stress [58, 59]. Clinicians in our study shared experiences around moral injury and burnout, and participating in the CLG improved self-reported resilience and decreased burnout. Thus, our study suggests that incorporating TIC in clinical practice may be bolstered by also addressing clinician wellbeing and burnout. Research indicates that clinician burnout is associated with reduced effectiveness of trauma-focused interventions [60], highlighting the critical importance of supporting clinician wellbeing to maintain high-quality TIC, particularly in sexual and reproductive health settings. In our study, clinicians shared moral conflict regarding sexual and reproductive healthcare such as options counseling, particularly when there was a difference in personal values between themselves and their patient, such as religious, moral, or otherwise. Focusing on empowering the patient to make their own decisions with the support of clinicians may prevent moral distress related to burnout from trauma-inquiry. To sustain effective trauma-informed practices, healthcare organizations can prioritize clinician well-being through regular training on trauma-informed care, trauma-informed systems, self-care and collective-care strategies such as clinician group debriefs [61]. We also recommend providing adequate staffing and resources (e.g., social work, legal resources) to manage complex patient needs, offering supervision and peer support for processing challenging cases, and implementing organizational policies that promote wellbeing and prevent burnout. These resources may also be considered when implementing TRIADS in other settings such as carceral and street medicine, where there is a high burden of patient trauma [62, 63]. Integrating education, skills development, and clinician support is a potential avenue for promoting effective patient care and clinician wellbeing simultaneously.

#### Limitations

There are limitations to this study. First, our narrative literature review approach was not systematic, and it is possible that we missed articles using this strategy. With a practical focus in mind, we chose to use the 43 articles that resulted from the narrative literature search in conjunction with an environmental scan to inform the development of our discussion-based training. Second, this study is limited to California and our findings may only be applied in the context of California state health policies. Finally, the attrition rate from the initial webinar to the CLGs was high; of 117 webinar participants, 52 marked interest in a follow-up discussion training, yet only 15 total individuals (some from the webinar, others from outside recruitment) participated in the CLGs. This is likely due to busy and unpredictable clinician schedules. Despite this, the intention of the CLGs was to foster an intimate, small-group discussion and our small sample size reached thematic saturation, in accordance with the literature [64].

## Conclusion

Trauma inquiry and response are key components of providing sexual and reproductive healthcare. Sexual and reproductive health services can improve health outcomes for trauma survivors by addressing the specific needs of this patient population, adapting trauma-informed approaches to time-limited encounters, focusing on relevant forms of trauma, navigating legal requirements, and supporting clinician well-being and the patient-clinician relationship.

Our virtual, live, discussion- and case-based training provided an opportunity for clinicians to share and learn TIC practices as well as to debrief experiences caring for patients with trauma histories. Our findings show that applying the TRIADS framework to sexual and reproductive health settings was effective in increasing clinician comfort in trauma inquiry and response. Future research should focus on developing and evaluating trauma-informed interventions specifically tailored to sexual and reproductive health contexts, as well as exploring innovative and sustainable strategies for promoting clinician wellbeing.

## Supplementary Information

Supplementary Material 1.

Supplementary Material 2.

Supplementary Material 3.

Supplementary Material 4.

## Data Availability

The datasets used and/or analyzed during the current study are available from the corresponding author on reasonable request.

## Abbreviations

ACEs: Adverse Childhood Experiences
IPV: Intimate Partner Violence
TRIADS: Trauma and Resilience-informed Inquiry for Adversity, Distress, and Strengths
CLG: Collaborative Learning Group
COREQ: Consolidated Criteria for Reporting Qualitative Research
TIC: Trauma-Informed Care
USPSTF: United States Preventive Services Task Force
STI: sexually transmitted infections

## References

[1] Hughes K, Bellis MA, Hardcastle KA, Sethi D, Butchart A, Mikton C, et al. The effect of multiple adverse childhood experiences on health: a systematic review and meta-analysis. Lancet Public Health. 2017;2:e356–66. 29253477 10.1016/S2468-2667(17)30118-4

[2] Bonomi AE, Anderson ML, Rivara FP, Thompson RS. Health outcomes in women with physical and sexual intimate partner violence exposure. J Women’s Health. 2007;16:987–97. 10.1089/jwh.2006.0239 17903075

[3] Ullman SE, Brecklin LR. Sexual assault history and Health-Related outcomes in a National sample of women. Psychol Women Q. 2003;27:46–57.

[4] Flaviano M, Harville EW. Adverse childhood experiences on reproductive plans and adolescent pregnancy in the Gulf resilience on women’s health cohort. Int J Environ Res Public Health. 2020;18:165. 33379385 10.3390/ijerph18010165 PMC7794759

[5] Huber-Krum S, Miedema SS, Shortt JW, Villaveces A, Kress H. Associations between adverse childhood experiences and contraceptive use among young adults in Honduras. Child Abuse Negl. 2022;123:105381. 34753054 10.1016/j.chiabu.2021.105381 PMC9511159

[6] Novick AM, Johnson RL, Lazorwitz A, Belyavskaya A, Berkowitz L, Norton A, et al. Discontinuation of hormonal contraception due to changes in mood and decreases in sexual desire: the role of adverse childhood experiences. Eur J Contracept Reprod Health Care. 2022;27:212–20. 35133231 10.1080/13625187.2022.2030702 PMC9133050

[7] Atzl VM, Narayan AJ, Rivera LM, Lieberman AF. Adverse childhood experiences and prenatal mental health: type of aces and age of maltreatment onset. J Fam Psychol. 2019;33:304–14. 30802085 10.1037/fam0000510

[8] Spencer CN, Khalil M, Herbert M, Aravkin AY, Arrieta A, Baeza MJ. Health effects associated with exposure to intimate partner violence against women and childhood sexual abuse: a burden of proof study. Nat Med. Dec; 2023;29(12):3243–58. 38081957 10.1038/s41591-023-02629-5 PMC10719101

[9] Calvillo C, Marshall A, Gafford S, Montgomery BEE. Intimate partner violence and its relation to sexual health outcomes across different adult populations: a systematic review. Available from: https://www.frontiersin.org/journals/sociology/articles/10.3389/fsoc.2024.1498969/full. 10.3389/fsoc.2024.1498969 PMC11671811 39735614

[10] Sarkar NN. The impact of intimate partner violence on women’s reproductive health and pregnancy outcome. J Obstet Gynaecol. Apr; 2008;28(3):266–71. 18569465 10.1080/01443610802042415

[11] Golding JM. Sexual assault history and women’s reproductive and sexual health. Psychol Women Q. 1996;1(1):101–21. 10.1111/j.1471-6402.1996.tb00667.x 12296010

[12] Hasstedt K, Review RAGP. Understanding Intimate Partner Violence as a Sexual and Reproductive Health and Rights Issue in the United States | Guttmacher Institute. 2016. Available from: https://www.guttmacher.org/gpr/2016/07/understanding-intimate-partner-violence-sexual-and-reproductive-health-and-rights-issue.

[13] Caring for Patients Who Have Experienced Trauma. Am Coll Obstetricians Gynecologists. 2021;No 825:e94–9.

[14] Hargrave AS, Ong A, Raza Z, Franco C, Castro AE, Nickols S. Opportunities for improving provision of emergency contraception in California. Contraception. 2022. 10.1016/j.contraception.2022.03.024 35381258

[15] DeMaria AL, Meier S, King H, Sidorowicz H, Seigfried-Spellar KC, Schwab-Reese LM. The role of community healthcare professionals in discussing sexual assault experiences during obstetrics and gynecological healthcare appointments. BMC Womens Health. 2023;23:263. 10.1186/s12905-023-02401-4 PMC10184971 37189119

[16] US Preventive Services Task Force, Curry SJ, Krist AH, Owens DK, Barry MJ, Caughey AB, et al. Screening for intimate partner violence, elder abuse, and abuse of vulnerable adults: US preventive services task force final recommendation statement. JAMA. 2018;320:1678. 30357305 10.1001/jama.2018.14741

[17] Owens L, Terrell S, Low LK, Loder C, Rhizal D, Scheiman L, et al. Universal precautions: the case for consistently trauma-informed reproductive healthcare. Am J Obstet Gynecol. 2022;226:671–7. 34418349 10.1016/j.ajog.2021.08.012

[18] SAMHSA’s Concept of Trauma and Guidance for a Trauma-Informed Approach. Rockville, MD: Substance Abuse and Mental Health Services Administration. 2014. Report No.: HHS Publication No. (SMA) 14-4884. Available from: https://library.samhsa.gov/product/samhsas-concept-trauma-and-guidance-trauma-informed-approach/sma14-4884.

[19] Terrell S, Mehari K, Oshman L, Otto AK, Ruff A, Owens L. Trauma-Informed pelvic examination practices among clinicians [A286]. Obstetrics & Gynecology. 2022;139(1):82S–3S.

[20] Nagle-Yang S, Sachdeva J, Zhao LX, Shenai N, Shirvani N, Worley LLM. Trauma-Informed care for obstetric and gynecologic settings. Matern Child Health J. Dec; 2022;26(12):2362–9. 36346563 10.1007/s10995-022-03518-y

[21] DeAndrade S, Pelletier A, Bartz D, Dutton C. Trauma informed care training in ob/gyn residency programs [26G]. Obstetrics & Gynecology. 2020;135:77S.

[22] Geiderman JM, Marco CA. Mandatory and permissive reporting laws: obligations, challenges, moral dilemmas, and opportunities. J Am Coll Emerg Physicians Open. 2020;1(1):38–45. 10.1002/emp2.12011 PMC7493571 33000012

[23] The TRIADS Framework. UCSF Center for Advance Trauma Informed Health Care. Available from: https://cthc.ucsf.edu/triads/. Cited 2023 Feb 27.

[24] Creswell J, Plano C. Desgining and conducting mixed methods research. Thousand Oaks, California: SAGE; 2018.

[25] Tong A, Sainsbury P, Craig J. Consolidated criteria for reporting qualitative research (COREQ): a 32-item checklist for interviews and focus groups. Int J Qual Health Care. 2007;1(6):349–57. 10.1093/intqhc/mzm042 17872937

[26] Shankar M, Loeliger K. Trauma Inquiry and Response in Family Planning. California, USA; 2023 [cited 2025 May 21]. Available from: https://familypact.org/resources/trauma-inquiry-and-response-in-family-planning/

[27] Zoom Video Communications, Inc. 2023. Available from: https://zoom.us.

[28] Onwuegbuzie AJ, Dickinson WB, Leech NL, Zoran AG. A qualitative framework for collecting and analyzing data in focus group research. Int J Qualitative Methods. 2009;8:1–21.

[29] Bingham AJ. From data management to actionable findings: A Five-Phase process of qualitative data analysis. Int J Qualitative Methods. 2023;22:16094069231183620.

[30] Dedoose. 2023. Available from: https://www.dedoose.com/

[31] Johnson S, Kasparian NA, Cullum AS, Flanagan T, Ponting C, Kowalewski L. Addressing adverse childhood and adult experiences during prenatal care. Obstet Gynecol. Jun; 2023;141(6):1072–87. 37141600 10.1097/AOG.0000000000005199 PMC10184824

[32] Palmieri J, Valentine JL. Using Trauma-Informed care to address sexual assault and intimate partner violence in primary care. J Nurse Practitioners. 2021;1(1):44–8.

[33] Lansing AE, Romero NJ, Siantz E, Silva V, Center K, Casteel D. Building trust: leadership reflections on community empowerment and engagement in a large urban initiative. BMC Public Health. 2023;23:1252. 10.1186/s12889-023-15860-z PMC10304359 37380973

[34] Ashworth H, Lewis-O’Connor A, Grossman S, Brown T, Elisseou S, Stoklosa H. Trauma-informed care (TIC) best practices for improving patient care in the emergency department. Int J Emerg Med. 2023;16(1):38. 10.1186/s12245-023-00509-w PMC10197231 37208640

[35] Kennedy AB, Harb AT, Schockling C, Ray LJ, Palomo J, Russ-Sellers R. Understanding the values, qualities, and preferences of patients in their relationships with obstetrics and gynecology providers: Cross-Sectional survey with a mixed methods approach. J Particip Med. 2024;16:16:e58096. 39412870 10.2196/58096 PMC11525076

[36] Fritz Z, Holton R. Too much medicine: not enough trust? J Med Ethics. 2019;1(1):31–5. 10.1136/medethics-2018-104866 PMC6327867 30367013

[37] Tervalon M, Murray-García J. Cultural humility versus cultural competence: a critical distinction in defining physician training outcomes in multicultural education. J Health Care Poor Underserved. 1998;9:117–25. 10073197 10.1353/hpu.2010.0233

[38] Curtis KM, Nguyen AT, Tepper NK, Zapata LB, Snyder EM, Hatfield-Timajchy K et al. U.S. Selected Practice Recommendations for Contraceptive Use, 2024;73. MMWR Recomm Rep. 2024. Available from: https://www.cdc.gov/mmwr/volumes/73/rr/rr7303a1.htm. Cited 2025 Feb 14. 10.15585/mmwr.rr7303a1 PMC11340200 39106301

[39] Machtinger EL, Lieberman AF, Bethell CD, Lightfoot M. Primary care as a protective factor: A vision to transform health care delivery and overcome disparities in health. Permanente J. 2024;28:193–7. 10.7812/TPP/23.109 PMC10940228 38361459

[40] Brown CK, DiBiase J, Nathanson A, Cadet TJ. Trauma-Informed care for inpatient palliative care social work: applying existing models at the bedside. J Soc Work End Life Palliat Care. 2023;19:309–25. 37698906 10.1080/15524256.2023.2256479 PMC10840610

[41] Goldstein E, Chokshi B, Melendez-Torres G, Rios A, Jelley M, Lewis-O’Connor A. Effectiveness of Trauma-Informed care implementation in health care settings. Syst Rev Reviews Realist Synthesis Perm J. 2024;28(1):135–50. 10.7812/TPP/23.127 PMC10940237 38444328

[42] Leblanc NM, Alexander K, Carter S, Crean H, Ingram L, Kobie J. The effects of trauma, violence. And stress on sexual health outcomes among female clinic clients in a small Northeastern. US Urban Center Womens Health Rep. New Rochelle. 2020. 10.1089/whr.2019.0027 PMC7325490 32617533

[43] Parrish M, Ryan S, Farone J. Negative reproductive health outcomes among adolescent females following sexual abuse, rape or assault. † 30. Pediatr Res. Apr; 1996;39(4):7–7.

[44] Salihu HM, August EM, Salemi JL, Weldeselasse H, Sarro YS, Alio AP. The association between female genital mutilation and intimate partner violence. BJOG. Dec; 2012;119(13):1597–605. 22925207 10.1111/j.1471-0528.2012.03481.x

[45] Sheeran N, Jenkins A, Humphreys T, Ter Horst S, Higgins M. Investigating the impact of reproductive coercion and intimate partner violence on psychological and sexual wellbeing. J Interpers Violence. Feb; 2025;40(3–4):726–55. 38752449 10.1177/08862605241253026 PMC11673295

[46] Reproductive and Sexual Coercion. Am Coll Obstetricians Gynecologists. 2022;No 554:411–5.

[47] Grace KT, Holliday CN, Bevilacqua K, Kaur A, Miller J, Decker MR. Sexual and reproductive health and reproductive coercion in women victim/survivors receiving housing support. J Fam Violence. 2023;38:713–22. 35283554 10.1007/s10896-022-00362-0 PMC8901387

[48] Maxwell L, Devries K, Zionts D, Alhusen JL, Campbell J. Estimating the effect of intimate partner violence on women’s use of contraception: A systematic review and Meta-Analysis. PLOS ONE. 2015;10(2):e0118234. 10.1371/journal.pone.0118234 PMC4334227 25693056

[49] Alspaugh A, Barroso J, Reibel M, Phillips S. Women’s contraceptive perceptions, beliefs, and attitudes. Integr Rev Qualitative Res J Midwifery Women’s Health. 2020;65:64–84. 10.1111/jmwh.12992 31135081

[50] Bleil ME, Adler NE, Pasch LA, Sternfeld B, Reijo-Pera RA, Cedars MI. Adverse childhood experiences and repeat induced abortion. Am J Obstet Gynecol. 2011;204(2):122.e1–6 . 10.1016/j.ajog.2010.09.029 PMC3032830 21074137

[51] Haddad S, Martin-Marchand L, Lafaysse M, Saurel-Cubizolles MJ. Repeat induced abortion and adverse childhood experiences in aquitaine, france: a cross-sectional survey. Eur J Contracept Reprod Health Care. 2021;26(1):29–35. 10.1080/13625187.2020.1815697 32914679

[52] Swift A, Berry M, Fernandez-Pineda M, Haberstroh A. An integrative review of adverse childhood experiences and reproductive traumas of infertility and pregnancy loss. J Midwifery Women’s Health. 2024;69:258–78. 38013638 10.1111/jmwh.13585

[53] Rabin RF, Jennings JM, Campbell JC, Bair-Merritt MH. Intimate partner violence screening tools. Am J Prev Med. 2009;36(5):439–45.e4 . 10.1016/j.amepre.2009.01.024 PMC2688958 19362697

[54] Madigan S, Deneault A, Racine N, Park J, Thiemann R, Zhu J. Adverse childhood experiences: a meta-analysis of prevalence and moderators among half a million adults in 206 studies. World Psychiatry. Oct; 2023;22(3):463–71. 37713544 10.1002/wps.21122 PMC10503911

[55] Justice CRDD. Title IX of the Education Amendments of 1972. Civil Rights, Division, U.S. Department of Justice. 2015. Available from: https://www.justice.gov/crt/title-ix-education-amendments-1972.

[56] Forouzan K, Guarnieri I, Fairbanks M, Curhan T, State Policy T. 2024: Anti-Abortion Policymakers Redouble Attacks on Bodily Autonomy. Guttmacher Institute. 2024. Available from: https://www.guttmacher.org/2024/12/state-policy-trends-2024-anti-abortion-policymakers-redouble-attacks-bodily-autonomy.

[57] Jusic B, Pilav A, Dzehverovic M, Cakar J. Analysis of aborted fetal material using autosomal STR markers in forensic cases of sexual assault. J Forensic Leg Med. 2023;94:102468. 10.1016/j.jflm.2022.102468 36584611

[58] Helpingstine C, Kenny MC, Malik F. Vicarious traumatization and burnout among service providers for victims of commercial sexual exploitation. J Child Sex Abuse. 2021;18(6):722–45. 10.1080/10538712.2021.1938771 34137346

[59] Baird S, Jenkins S. Vicarious traumatization, secondary traumatic stress, and burnout in sexual assault and domestic violence agency staff. Violence Vict. Feb; 2003;18(1):71–86. 12733620 10.1891/vivi.2003.18.1.71

[60] Sayer NA, Kaplan A, Nelson DB, Wiltsey Stirman S, Rosen CS. Clinician burnout and effectiveness of Guideline-Recommended psychotherapies. JAMA Netw Open. 2024;17(4):e246858. 10.1001/jamanetworkopen.2024.6858 PMC11024738 38630477

[61] Kim J, Chesworth B, Franchino-Olsen H, Macy RJ. A scoping review of vicarious trauma interventions for service providers working with people who have experienced traumatic events. Trauma Violence Abuse. Dec; 2022;23(5):1437–60. 33685294 10.1177/1524838021991310 PMC8426417

[62] Forman-Dolan J, Caggiano C, Anillo I, Kennedy TD. Burnout among professionals working in corrections: A two stage review. Int J Environ Res Public Health. 2022;19:9954. 36011590 10.3390/ijerph19169954 PMC9408353

[63] Ng L, Adams E, Henderson D, Donaghy E, Mercer SW. Interventions for burnout and well-being in homelessness staff: a systematic scoping review. medRxiv. 2024 [cited 2025 May 21]. p. 2024.08.21.24312389. Available from: https://www.medrxiv.org/content/10.1101/2024.08.21.24312389v1 10.1371/journal.pone.0309866 PMC12094747 40397915

[64] Guest G, Namey E, Chen M. A simple method to assess and report thematic saturation in qualitative research. PLoS ONE. 2020;15:e0232076. 32369511 10.1371/journal.pone.0232076 PMC7200005

