# Supplementary material for: Trauma inquiry and response in sexual and reproductive health settings: collaborative learning among clinicians
Source: Reprod Health. 2025 Sep 29;22:164. doi: 10.1186/s12978-025-02135-6 (PMC12481804; doi:10.1186/s12978-025-02135-6)
Supplement: Supplementary file 1 — Supplementary Material 1. [file 12978_2025_2135_MOESM1_ESM.docx]

**Supplement 1. Post-Webinar Survey**

**CONSENT**

Thank you for participating in our webinar. This survey will take about 5 minutes to complete. Your participation is completely voluntary, and you can stop at any time. Your responses are confidential, and we will not be using any personal identifiers that would link you to your responses. Please answer these questions as honestly as possible as your responses will help guide trauma-related training in family planning.

- Do you consent to this survey?
  - Yes
  - No

**IMPLEMENTATION QUESTIONS**

- How likely are you to use the TRIADS framework in your family planning clinical practice?
- 1 = Very Unlikely
- 2 = Unlikely
- 3 = Likely
- 4 = Very Likely

- What are some reasons that it might be difficult to use the TRIADS framework in your family planning clinical practice? Select all that apply.
- Time constraints
- Lack of training
- Discomfort with asking patients about trauma
- Unsure how to respond to patient trauma
- Lack of referral resources
- Other reasons: *___________*

- What are some of the reasons that it might be easy to use the TRIADS framework in your family planning clinical practice? Select all that apply.
- Comfort with using a small amount of time during visits to discuss trauma
- Adequate training
- Comfort with asking patients about trauma
- Confidence in how to respond to patient trauma
- Adequate referral resources
- Other reasons: *___________*

- Are you interested in participating in a follow up training that will entail a 1-hour discussion with other family planning clinicians?
  - Yes
  - No

**DEMOGRAPHICS**

- What clinical training have you completed?
  - MD/DO
  - NP
  - CNM
  - PA
  - RN
  - MA
  - Other/Write-In: _______
- How many years have you been in clinical practice?
  - 1 – 5
  - 6 – 10
  - 11 – 20
  - 21 – 30
  - 30+
- What is your gender?
  - Man
  - Woman
  - Non-binary/Genderqueer
- What is your race/ethnicity (select all that apply):
  - Hispanic, Latino, or Spanish origin
  - Black or African American
  - East, Southeast, or South Asian
  - Native American or Alaska Native
  - Native Hawaiian or Pacific Islander
  - Middle Eastern or North African
  - White or European Origin
  - Other/Write-In: _______
- What is the best email address to contact you for study purposes? _________________________________
