# Supplementary material for: Trauma inquiry and response in sexual and reproductive health settings: collaborative learning among clinicians
Source: Reprod Health. 2025 Sep 29;22:164. doi: 10.1186/s12978-025-02135-6 (PMC12481804; doi:10.1186/s12978-025-02135-6)
Supplement: Supplementary file 2 — Supplementary Material 2. [file 12978_2025_2135_MOESM2_ESM.docx]

**Pre-CLG Survey**

Start of Block: CONSENT

Thank you for participating in the collaborative learning group! As a part of our study, we invite you to complete a brief pre-survey, which will take about 5 minutes to complete. Your participation is completely voluntary, and you can stop at any time. Your responses are confidential, and we will not be using any personal identifiers that would link you to your responses. Please answer these questions as honestly as possible as your responses will help guide trauma-related training in family practice.

Do you consent to this survey?

- Yes
- No

Skip To: End of Survey If Thank you for participating in the collaborative learning group! As a part of our study, we invit... = No

End of Block: CONSENT

Start of Block: Previous Patient Populations & Experiences

How often do you work with patients who have disclosed a history of trauma?

- Daily
- At least once per week
- At least once per month
- Rarely
- Never

How have you become aware of trauma your patients have experienced? (Select all that apply)

- Patient chart review
- Screening tool
- Patient's direct disclosure to you
- Patient's response to direct inquiry from you
- Your clinical suspicion based on history and/or exam
- Other (please specify): __________________________________________________

Approximately what proportion of the patients you see identify as cis-female?

- None
- Less than half
- About half
- Majority
- All of my patients identify as cis-female

Approximately what proportion of the patients you see identify as transgender and/or gender-diverse?

- None
- Less than half
- About half
- Majority
- All of my patients identify as transgender and/or gender-diverse

Do you provide healthcare for any of the following populations? (Select all that apply)

- People with substance use disorders
- People with unstable housing
- People with a past or current history of intimate partner violence
- People with a trauma related to being an immigrant, refugee, or asylum-seeker
- People with a known history of trauma of any kind
- Other types of patients (please specify): __________________________________________________

End of Block: Previous Patient Populations & Experiences

Start of Block: Trauma-Related Training

What motivated you to attend this training?

________________________________________________________________

Are there any specific patient encounters or scenarios that you would like to discuss? If so, please specify here:

________________________________________________________________

What training around trauma have you received in the past? (Select all that apply)

- No training
- Formal class/workshop training in school (Enter # of hours below) __________________________________________________
- Training class/workshop offered through your job (Enter # of hours below) __________________________________________________
- Informal training through webinar, articles, or other online resources
- Other (please specify): __________________________________________________

How comfortable are you with asking patients about past trauma?

- 1 - Very uncomfortable
- 2 - Uncomfortable
- 3 - Neither comfortable nor uncomfortable
- 4 - Comfortable
- 5 - Very comfortable

How comfortable are you with responding to patient’s disclosure of trauma?

- 1 - Very uncomfortable
- 2 - Uncomfortable
- 3 - Neither comfortable nor uncomfortable
- 4 - Comfortable
- 5 - Very comfortable

Have you received training regarding how to perform a trauma-informed pelvic exam?

- Yes
- No

How comfortable are you with performing a trauma-informed pelvic exam?

- 1 - Very uncomfortable
- 2 - Uncomfortable
- 3 - Neither comfortable nor uncomfortable
- 4 - Comfortable
- 5 - Very comfortable

How comfortable are you with applying TRIADS (Trauma and Resilience-informed Inquiry for Adversity, Distress, and Strengths) in your clinical setting and practice?

- 1 - Very uncomfortable
- 2 - Uncomfortable
- 3 - Neither comfortable nor uncomfortable
- 4 - Comfortable
- 5 - Very comfortable

End of Block: Trauma-Related Training

Start of Block: Demographics

What clinical training have you completed?

- MD/DO
- NP
- CNM
- PA
- RN
- MA
- Other (please specify): __________________________________________________
- Prefer not to answer

Which specialty are you most closely affiliated with?

- Obstetrics and Gynecology
- Midwifery
- Internal Medicine
- Family Medicine
- Preventive Medicine
- Pediatrics
- Other (please specify): __________________________________________________

How many years have you been in clinical practice?

- 1-5
- 6-10
- 11-20
- 21-30
- 30+
- Prefer not to answer

What gender do you identify as?

- Man
- Woman
- Non-binary/Genderqueer
- Other (please specify): __________________________________________________
- Prefer not to answer

What is your race/ethnicity? (Select all that apply)

- Hispanic, Latino, or Spanish origin
- Black or African American
- East, Southeast, or South Asian
- Native American or Alaska Native
- Native Hawaiian or Pacific Islander
- Middle Eastern or North African
- White or European origin
- Other (please specify): __________________________________________________
- Prefer not to answer

What type of facility do you work at? (Select all that apply)

- Federally qualified health center
- Community-based clinic
- Academic-affiliated clinic
- Veterans Affairs clinic
- Refugee health clinic
- Homeless health care clinic
- Planned Parenthood
- Other (please specify): __________________________________________________

Please enter the following contact information (ONLY to be used for study purposes):

- Email: __________________________________________________
- Confirm Email: __________________________________________________
- Last 4 digits of phone number: __________________________________________________

End of Block: Demographics

**Immediate Post-CLG Survey**

Thank you for participating in the collaborative learning group! As a part of our study, we invite you to complete a brief post-training survey, which will take about 5 minutes to complete. Your participation is completely voluntary, and you can stop at any time. Your responses are confidential, and we will not be using any personal identifiers that would link you to your responses. Please answer these questions as honestly as possible as your responses will help guide trauma-related training in family practice.

Do you consent to this survey?

- Yes
- No

Skip To: End of Survey If Thank you for participating in the collaborative learning group! As a part of our study, we invit... = No

End of Block: CONSENT

Start of Block: Trauma-Related Training

How comfortable are you with asking patients about past trauma?

- 1 - Very uncomfortable
- 2 - Uncomfortable
- 3 - Neither comfortable nor uncomfortable
- 4 - Comfortable
- 5 - Very comfortable

How comfortable are you with responding to patient’s disclosure of trauma?

- 1 - Very uncomfortable
- 2 - Uncomfortable
- 3 - Neither comfortable nor uncomfortable
- 4 - Comfortable
- 5 - Very comfortable

How comfortable are you with performing a trauma-informed pelvic exam?

- 1 - Very uncomfortable
- 2 - Uncomfortable
- 3 - Neither comfortable nor uncomfortable
- 4 - Comfortable
- 5 - Very comfortable

How comfortable are you with applying TRIADS (Trauma and Resilience-informed Inquiry for Adversity, Distress, and Strengths) in your clinical setting and practice?

- 1 - Very uncomfortable
- 2 - Uncomfortable
- 3 - Neither comfortable nor uncomfortable
- 4 - Comfortable
- 5 - Very comfortable

What feedback or suggestions do you have about this collaborative learning group experience?

________________________________________________________________

End of Block: Trauma-Related Training

Start of Block: Contact Info

Please enter the following contact information (ONLY to be used for study purposes):

- Email: __________________________________________________
- Confirm Email: __________________________________________________
- Last 4 digits of phone number: __________________________________________________

End of Block: Contact Info

**4-Month Post-CLG Survey**

Thank you for participating in the TRUST Collaborative Learning Group about 4 months ago! As a part of our study, we ask you to complete a brief, longitudinal post-survey, which will take about 5 minutes to complete. Your participation is completely voluntary, and you can stop at any time. Your responses are confidential, and we will not be using any personal identifiers that would link you to your responses. Please answer these questions as honestly as possible as your responses will help guide trauma-related training in family planning.

- Do you consent to this survey?
  - Yes
  - No
- Skip To: End of Survey If Thank you for participating in the collaborative learning group! As a part of our study, we invit... = No
- End of Block: CONSENT
- Start of Block: Trauma-Related Training
- How comfortable are you with asking patients about past trauma?
  - 1 – Very uncomfortable
  - 2 – Uncomfortable
  - 3 – Neither uncomfortable nor comfortable
  - 4 – Comfortable
  - 5 – Very comfortable
- How comfortable are you with responding to patient disclosure of trauma?
  - 1 – Very uncomfortable
  - 2 – Uncomfortable
  - 3 – Neither uncomfortable nor comfortable
  - 4 – Comfortable
  - 5 – Very comfortable
- How comfortable are you with performing a trauma informed pelvic exam?
  - 1 – Very uncomfortable
  - 2 – Uncomfortable
  - 3 – Neither uncomfortable nor comfortable
  - 4 – Comfortable
  - 5 – Very comfortable
- How comfortable are you with applying the TRIADS framework in your clinical setting and practice?
  - 1 – Very uncomfortable
  - 2 – Uncomfortable
  - 3 – Neither uncomfortable nor comfortable
  - 4 – Comfortable
  - 5 – Very comfortable
- Please describe any clinical practice changes related to trauma and resilience inquiry you have made since the TRUST Collaborative Learning Group: ________________________________________________________________________

- Please describe any changes you have made to support your resilience as a clinician and prevent burnout since the TRUST Collaborative Learning Group: ________________________________________________________________________
- How would you describe your feelings regarding your resilience as a clinician since the TRUST Collaborative Learning Group?
  - 1: Significantly increased resilience
  - 2: Increased resilience
  - 3: No change to resilience
  - 4: Decreased resilience
  - 5: Significantly decreased resilience
- How would you describe your experience of burnout as a clinician since the TRUST Collaborative Learning Group?
  - 1: Significantly decreased burnout
  - 2: Decreased burnout
  - 3: No change to burnout
  - 4: Increased burnout
  - 5: Significantly increased burnout
- End of Block: Trauma-Related Training
- Start of Block: Contact Info
- What is your email (only to be used for study purposes)? _______________________________
- What is the last 4 digits of your phone number (only to be used for study purposes)? _________
- End of Block: Contact Info
