# Supplementary material for: Trauma inquiry and response in sexual and reproductive health settings: collaborative learning among clinicians
Source: Reprod Health. 2025 Sep 29;22:164. doi: 10.1186/s12978-025-02135-6 (PMC12481804; doi:10.1186/s12978-025-02135-6)
Supplement: Supplementary file 3 — Supplementary Material 3. [file 12978_2025_2135_MOESM3_ESM.pptx]

## Slide 1
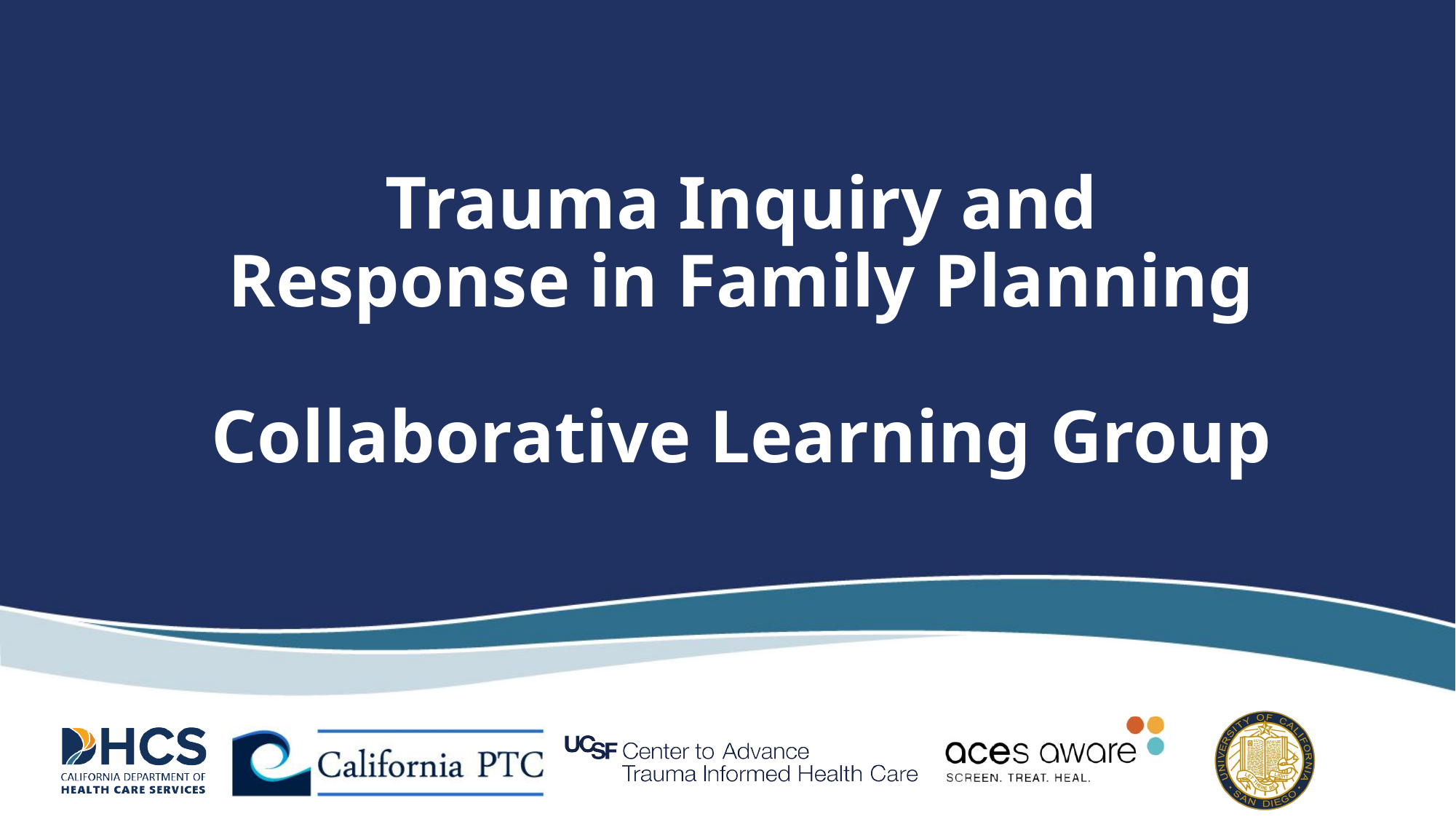

# Trauma Inquiry and Response in Family Planning Collaborative Learning Group

## Slide 2
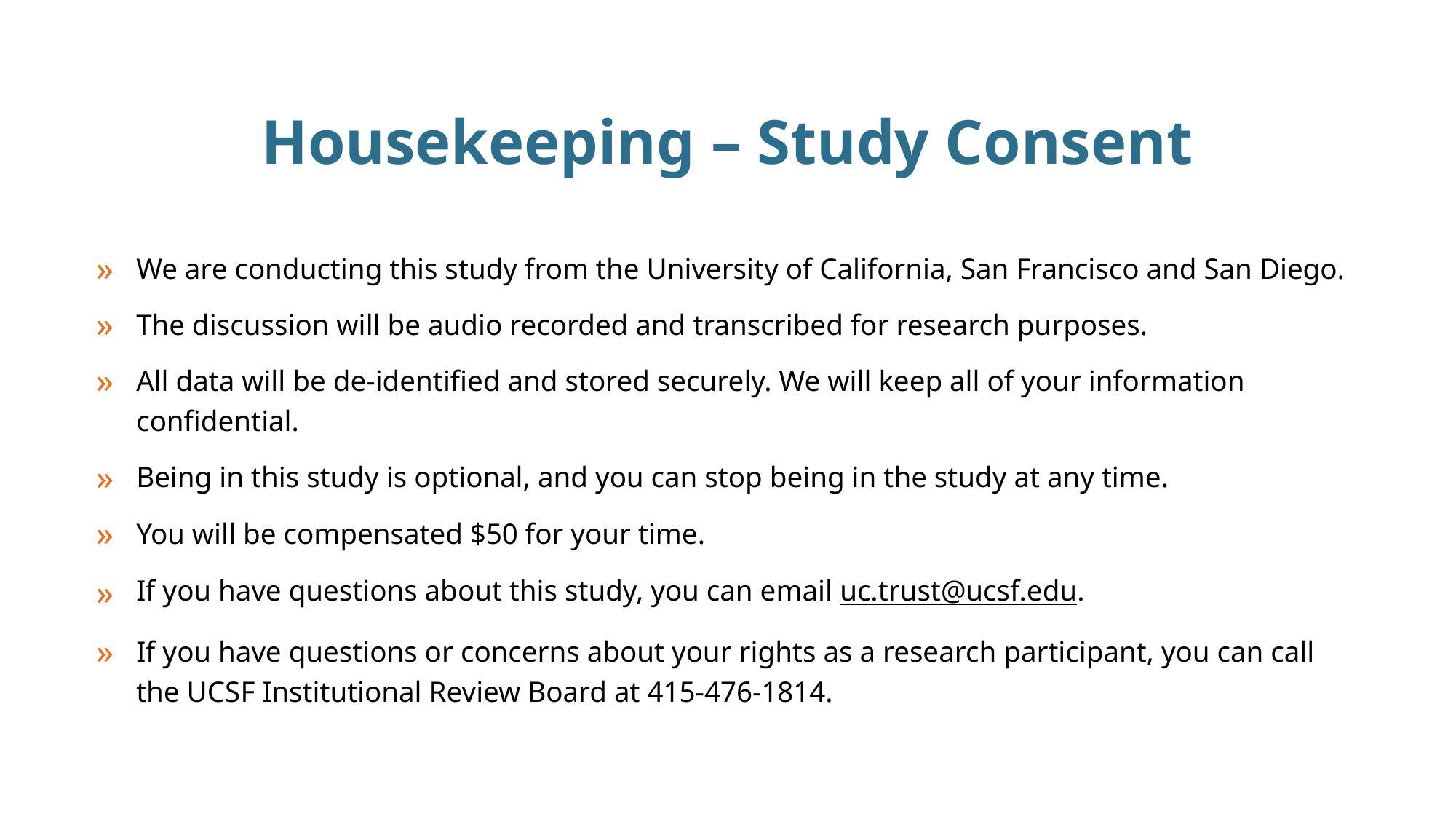

# Housekeeping – Study Consent
We are conducting this study from the University of California, San Francisco and San Diego.
The discussion will be audio recorded and transcribed for research purposes.
All data will be de-identified and stored securely. We will keep all of your information confidential.
Being in this study is optional, and you can stop being in the study at any time.
You will be compensated $50 for your time.
If you have questions about this study, you can email uc.trust@ucsf.edu.
If you have questions or concerns about your rights as a research participant, you can call the UCSF Institutional Review Board at 415-476-1814.

## Slide 3
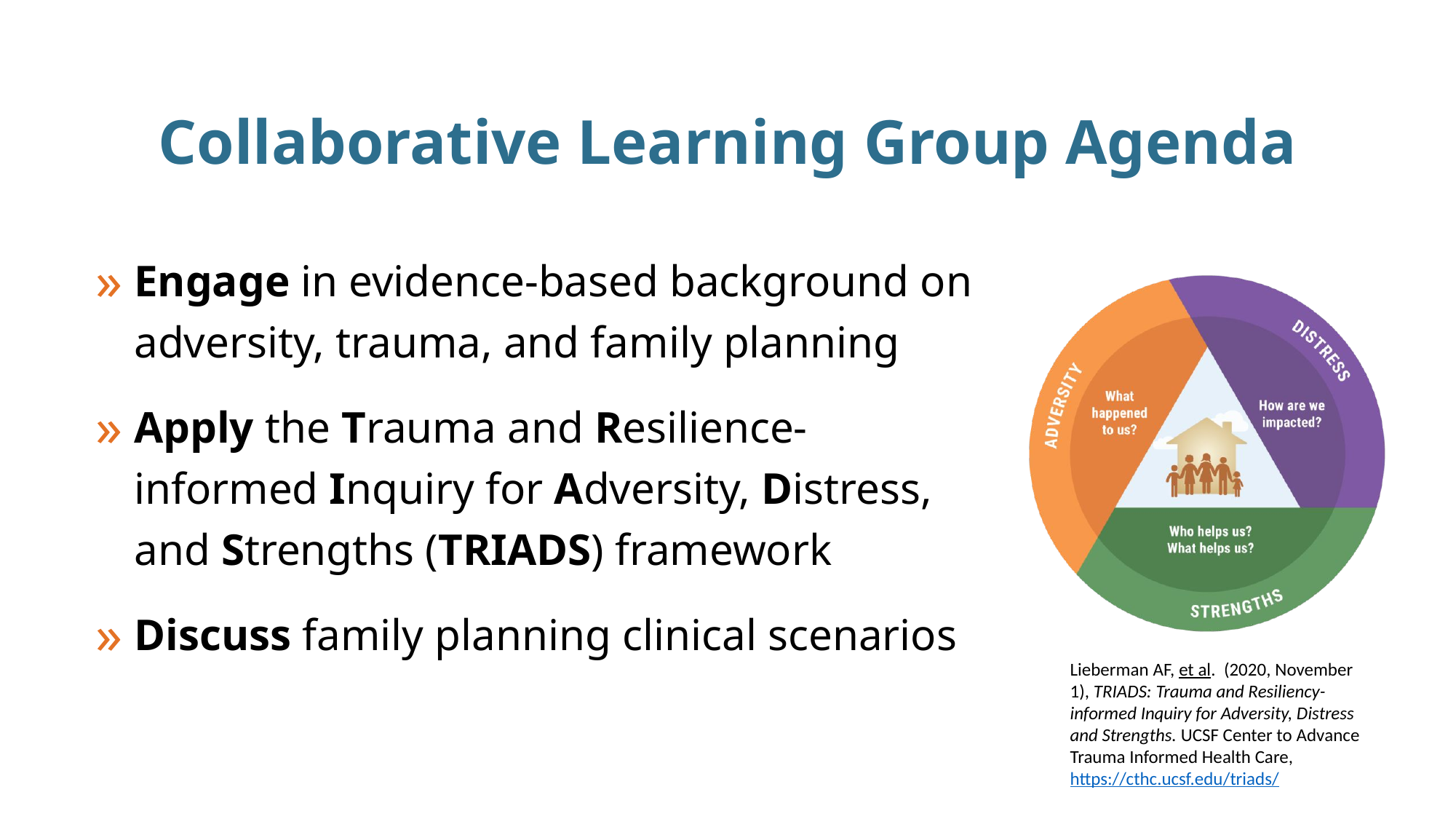

# Collaborative Learning Group Agenda
Engage in evidence-based background on adversity, trauma, and family planning
Apply the Trauma and Resilience-informed Inquiry for Adversity, Distress, and Strengths (TRIADS) framework
Discuss family planning clinical scenarios
Lieberman AF, et al.  (2020, November 1), TRIADS: Trauma and Resiliency-informed Inquiry for Adversity, Distress and Strengths. UCSF Center to Advance Trauma Informed Health Care,  https://cthc.ucsf.edu/triads/

## Slide 4
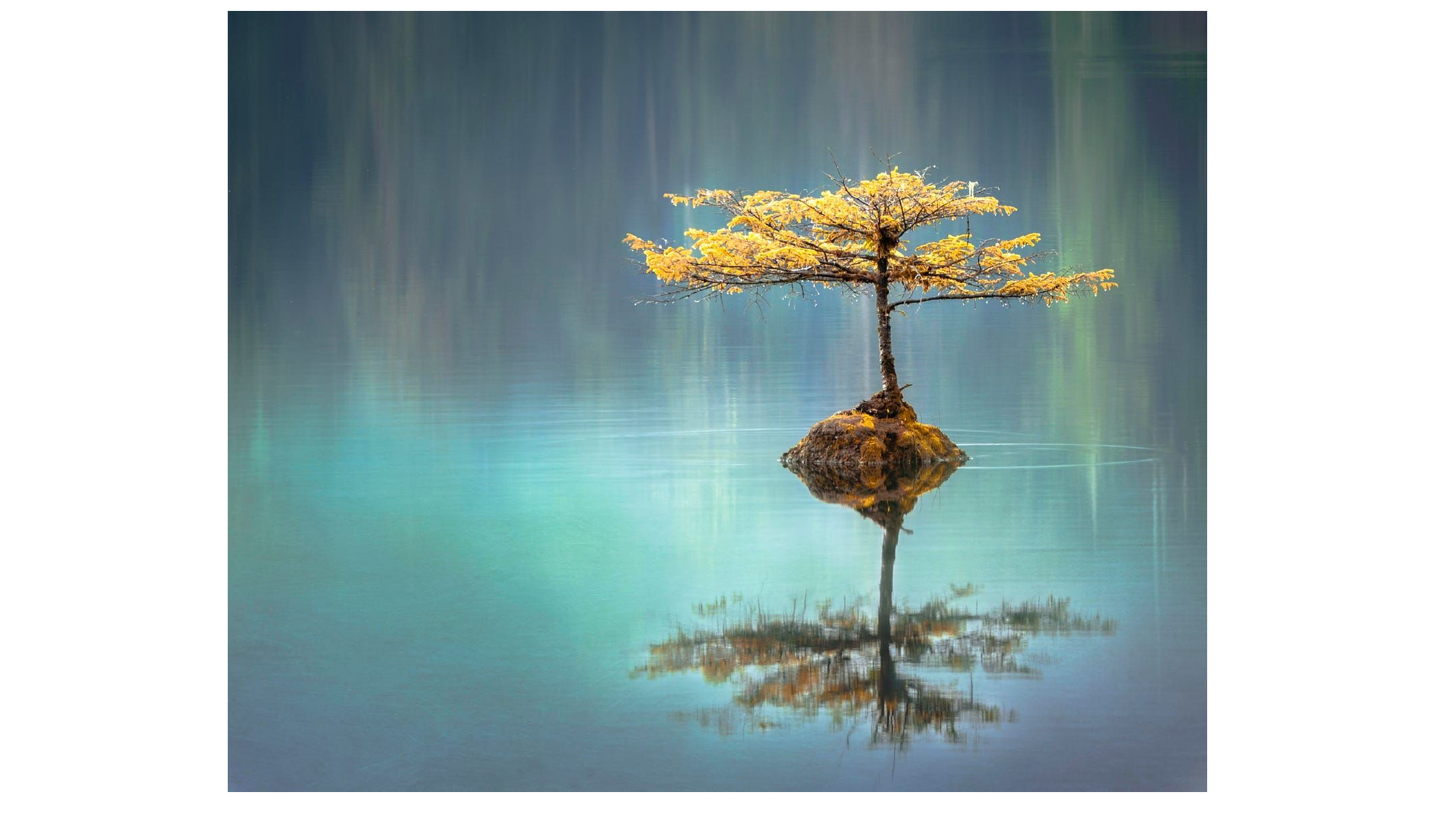

## Slide 5
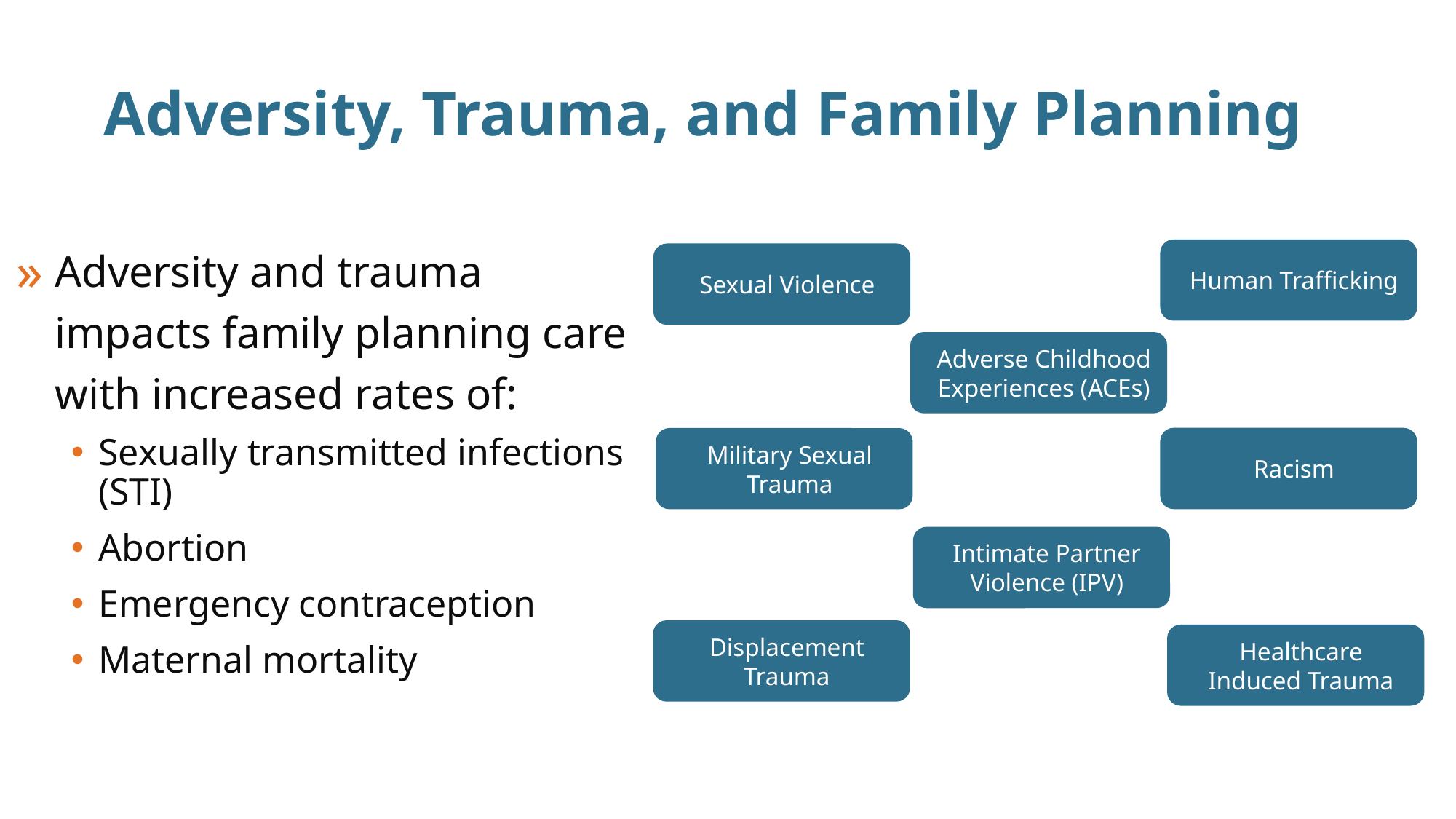

# Adversity, Trauma, and Family Planning
Adversity and trauma impacts family planning care with increased rates of:
Sexually transmitted infections (STI)
Abortion
Emergency contraception
Maternal mortality
Human Trafficking
Sexual Violence
Adverse Childhood Experiences (ACEs)
Racism
Military Sexual Trauma
Intimate Partner Violence (IPV)
Displacement Trauma
Healthcare Induced Trauma

## Slide 6
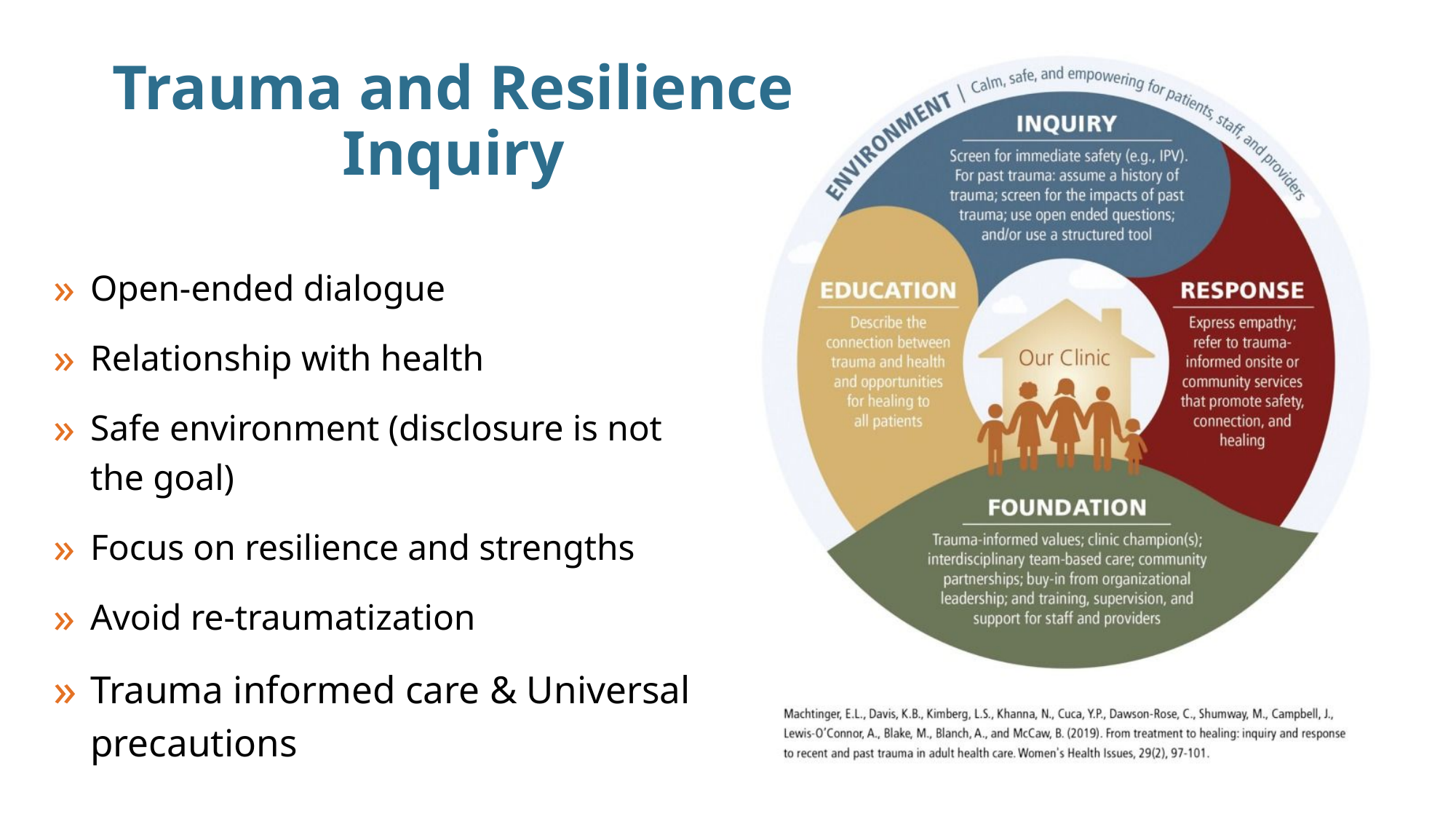

# Trauma and Resilience Inquiry
Open-ended dialogue
Relationship with health
Safe environment (disclosure is not the goal)
Focus on resilience and strengths
Avoid re-traumatization
Trauma informed care & Universal precautions

## Slide 7
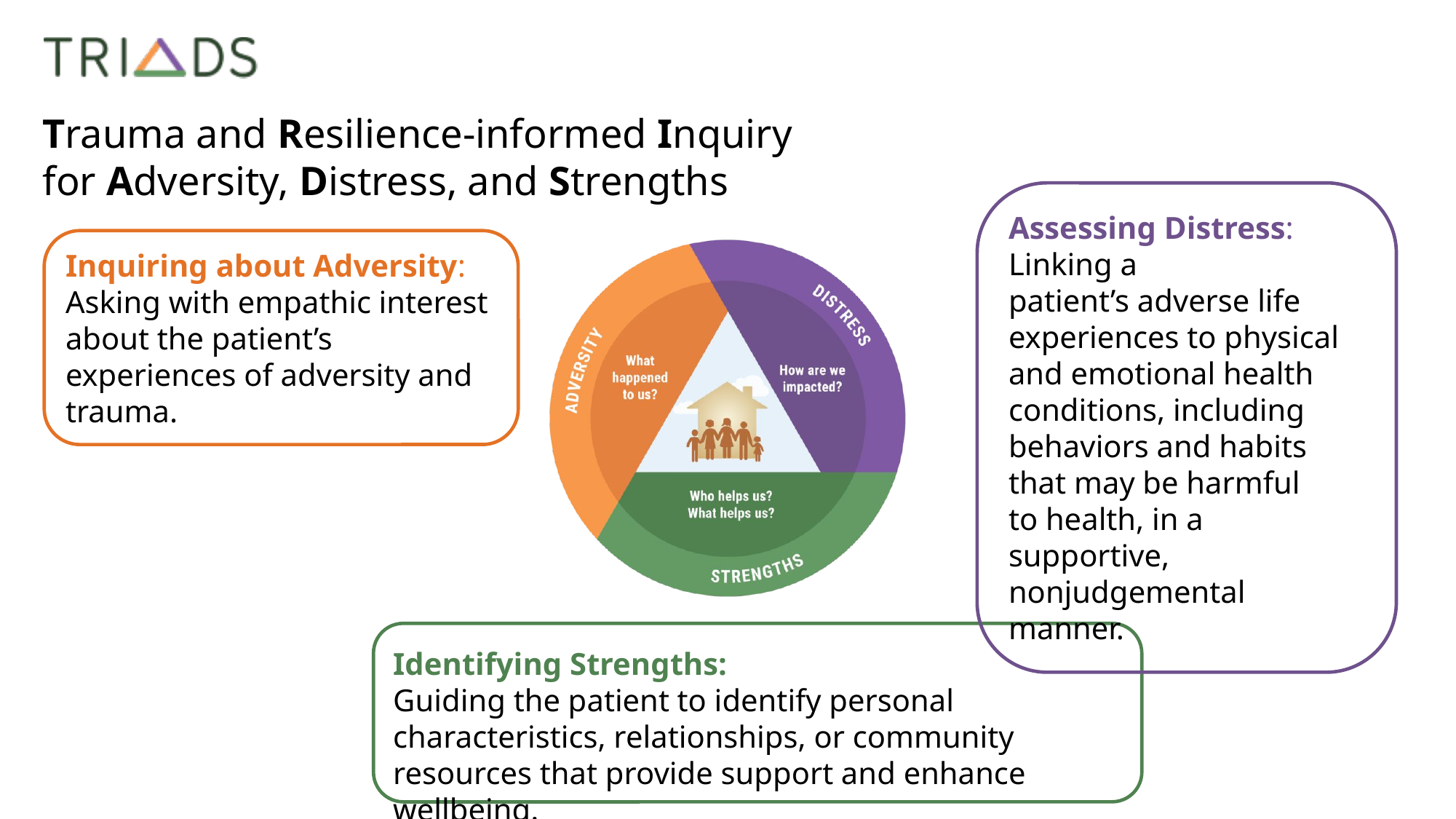

Trauma and Resilience-informed​ Inquiry for Adversity, Distress, and Strengths​
Assessing Distress:
Linking a patient’s adverse life experiences to physical and emotional health conditions, including behaviors and habits that may be harmful to health, in a supportive, nonjudgemental manner.
Inquiring about Adversity:
Asking with empathic interest about the patient’s experiences of adversity and trauma.
Identifying Strengths:
Guiding the patient to identify personal characteristics, relationships, or community resources that provide support and enhance wellbeing.

## Slide 8
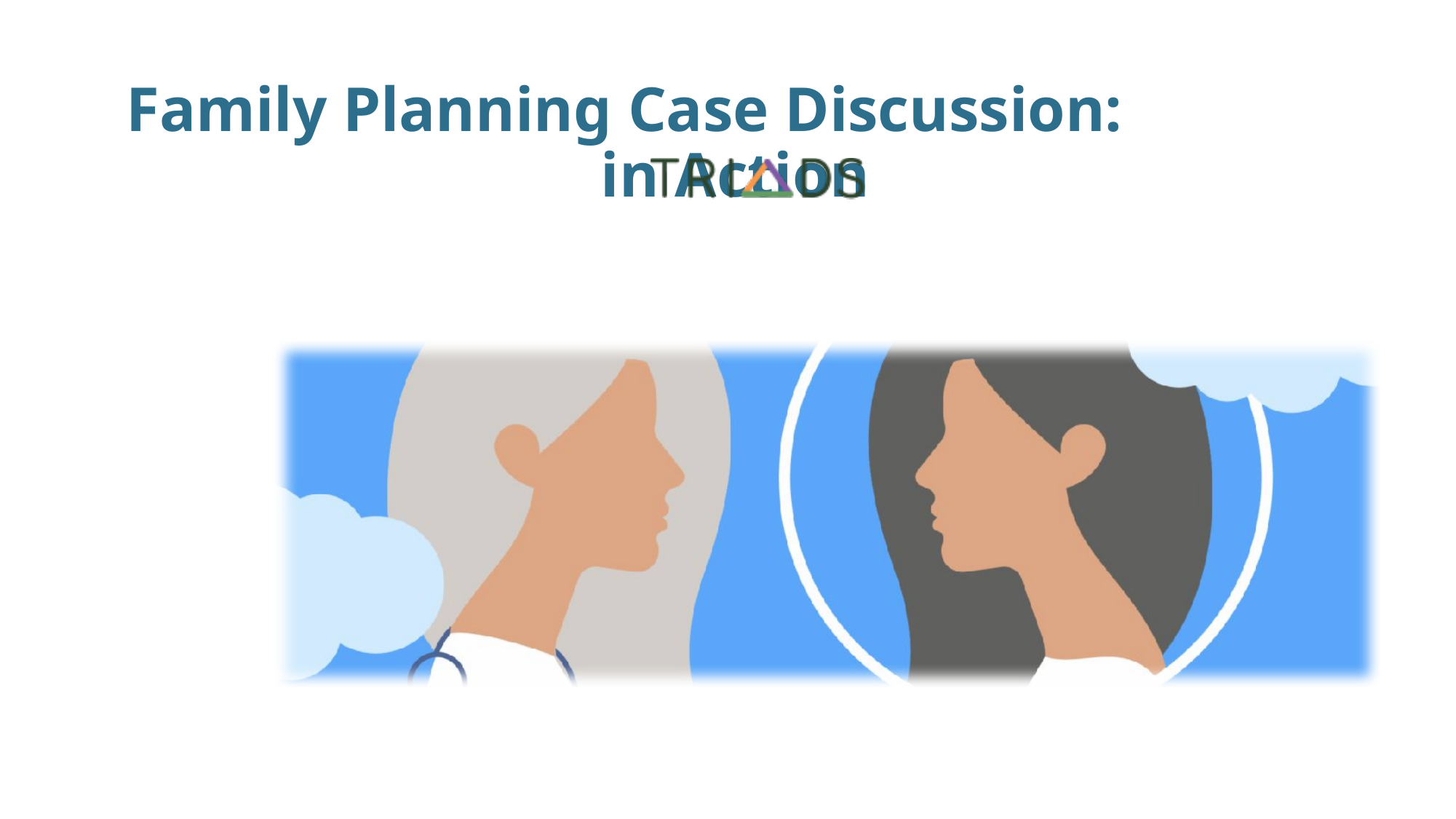

# Family Planning Case Discussion:               in Action

## Slide 9
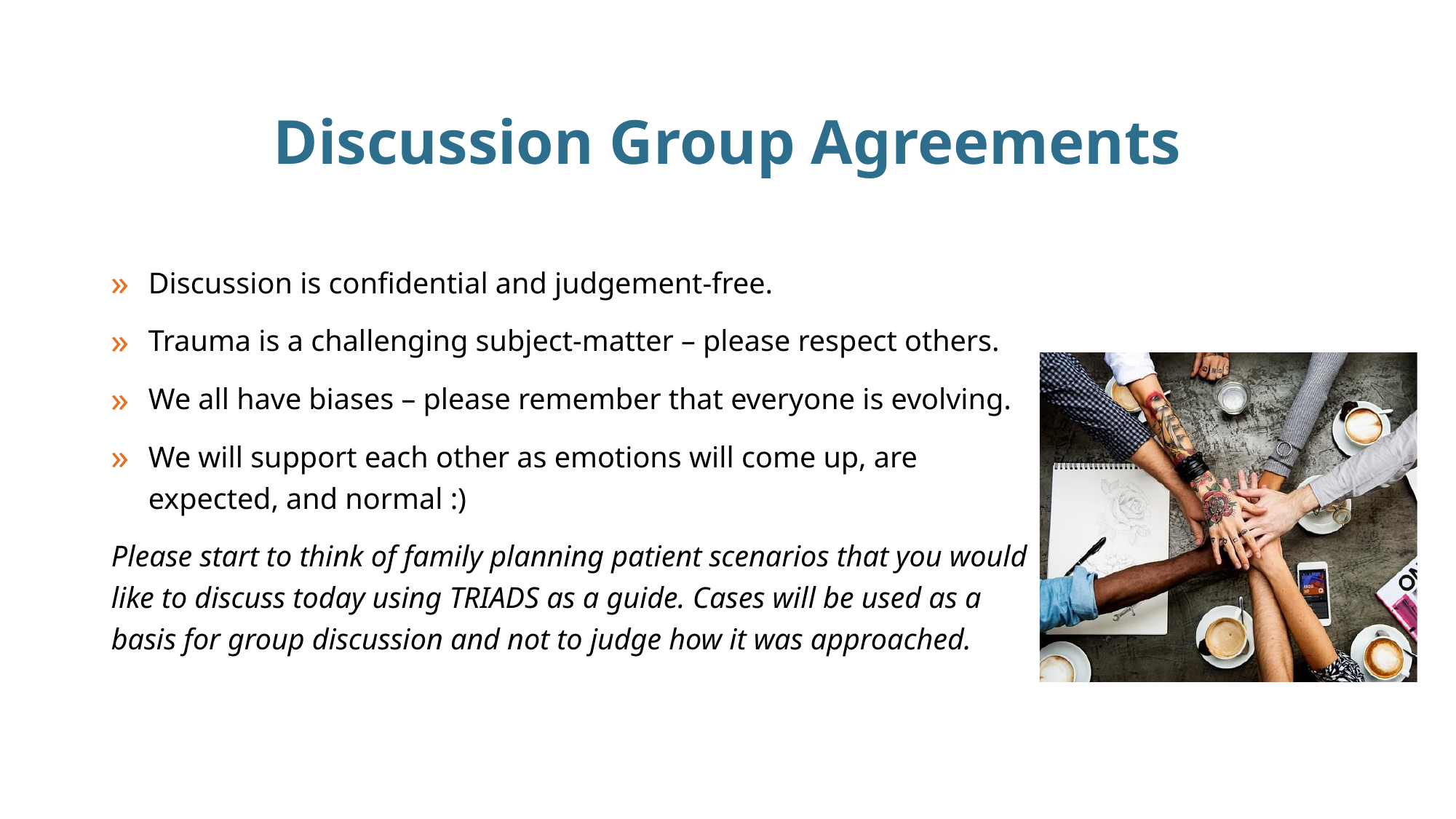

# Discussion Group Agreements
Discussion is confidential and judgement-free.
Trauma is a challenging subject-matter – please respect others.
We all have biases – please remember that everyone is evolving.
We will support each other as emotions will come up, are expected, and normal :)
Please start to think of family planning patient scenarios that you would like to discuss today using TRIADS as a guide. Cases will be used as a basis for group discussion and not to judge how it was approached.

## Slide 10
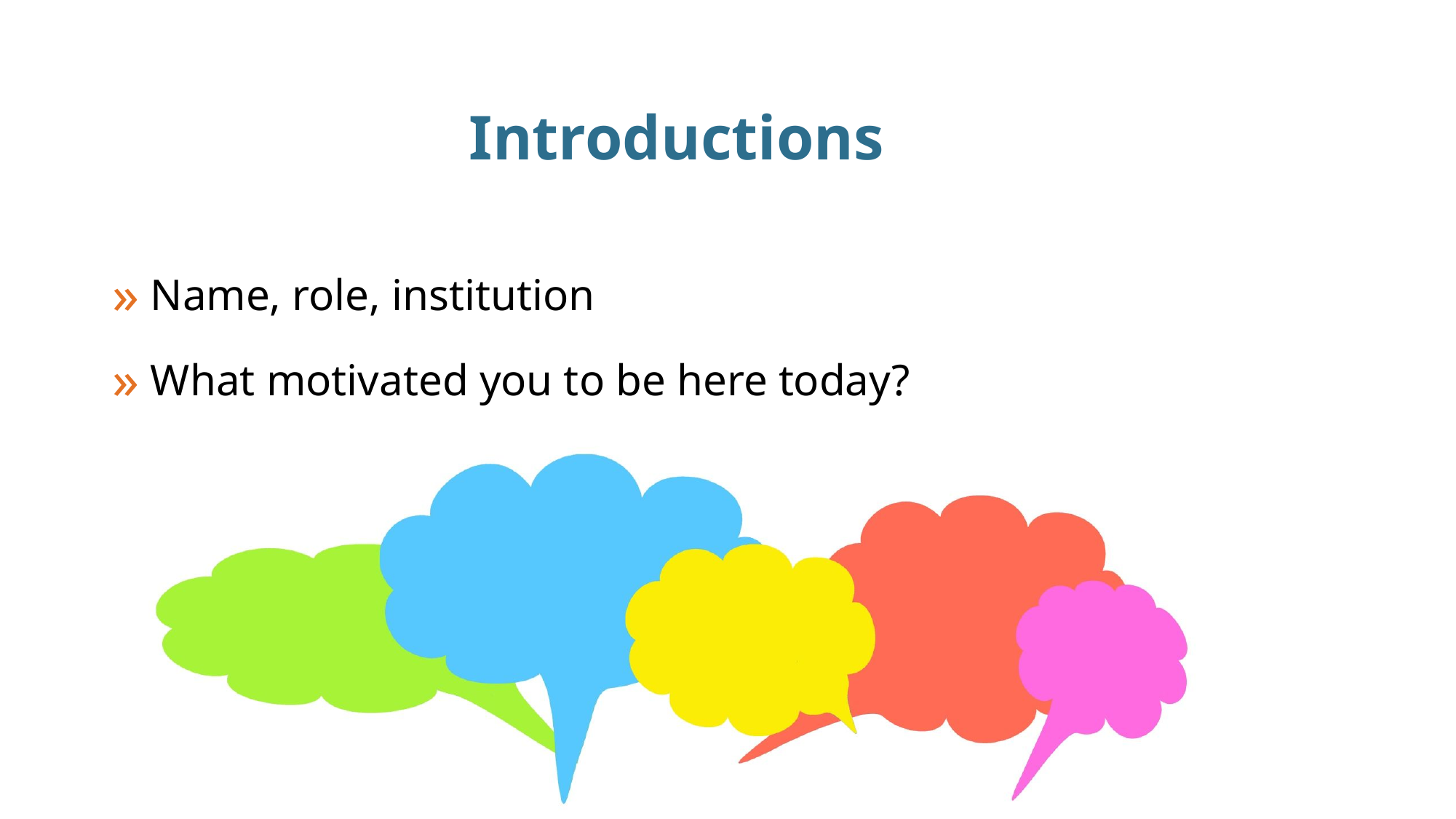

# Introductions
Name, role, institution
What motivated you to be here today?

## Slide 11
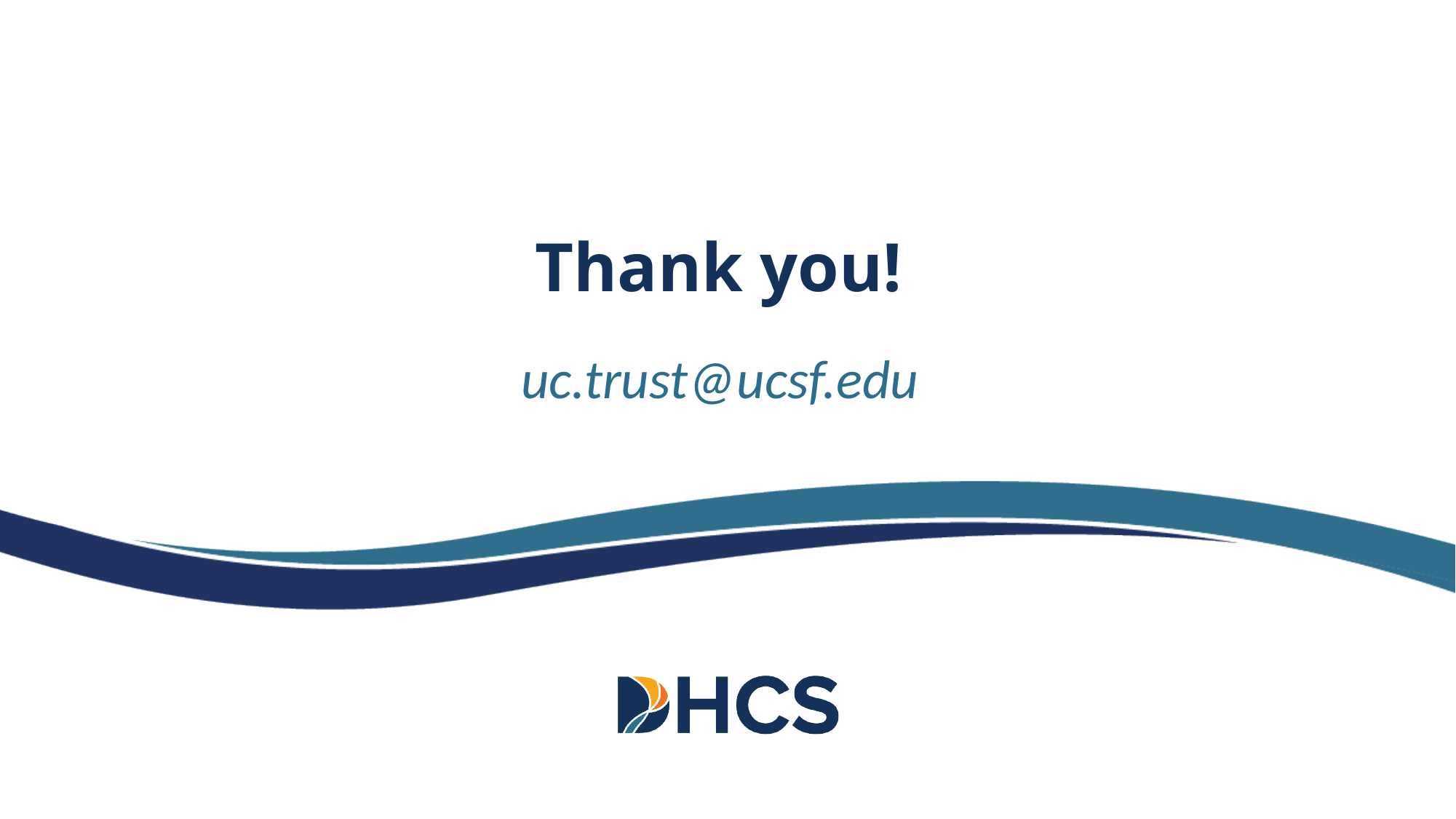

# Thank you!
uc.trust@ucsf.edu
