# Supplementary material for: Trauma inquiry and response in sexual and reproductive health settings: collaborative learning among clinicians
Source: Reprod Health. 2025 Sep 29;22:164. doi: 10.1186/s12978-025-02135-6 (PMC12481804; doi:10.1186/s12978-025-02135-6)
Supplement: Supplementary file 4 — Supplementary Material 4. [file 12978_2025_2135_MOESM4_ESM.docx]

**Collaborative Learning Group (CLG) Codebook**

| **Code** | **Description** |
| --- | --- |
| Adversity | Practices that clinicians use to ask with empathetic interest about patients’ experience of adversity or trauma. This can also include screening using validated tools either verbally or paper or online. |
| Distress | Practices that clinicians use to assess patient signs of distress. This also includes clinicians describing in a supportive, non-judgmental manner the possible links between the patient’s adverse life experiences and presenting physical and emotional health conditions associated with trauma, including behaviors and habits that may be harmful to health. |
| Strengths | Practices that clinicians use to identify patient strengths and resilience as a way to heal. This includes clinicians guiding the patient to identify personal characteristics, relationships, or community resources that provide support and enhance wellbeing. |
| Verbal | Specific words or phrases that clinicians use or recommend using to apply the TRIADS framework in family planning clinical practice. |
| Non-verbal | Non-verbal communication practices that clinicians use or recommend using to apply the TRIADS framework in family planning clinical practice. |
| Non-TRIADS | Practices that clinicians use that do not fall under adversity, distress, or strengths, but address trauma inquiry and response in family planning. |
| Trauma informed pelvic exam | Practices that clinicians use that are specific to conducting a trauma-informed pelvic exam. |
| TRIADS Facilitators | Practices that make it easier for clinicians to apply TRIADS in family planning settings. This can also include systemic facilitators (clinic-level practices) or interpersonal facilitators (individual-level practices such as self-care, resilience, and burnout prevention). |
| TRIADS Barriers | Practices that make it harder for clinicians to apply TRIADS in family planning settings. This can include systemic barriers (e.g., legal concerns around mandated reporting) or interpersonal barriers (e.g., experiences that lead to moral tension or secondary trauma). |
| Quotes | Exemplary quotes for the manuscript. |
